# Targeted Cepharanthine-Mediated Dual-Pathway Inhibition Sensitizes Pancreatic Cancer to Gemcitabine

**DOI:** 10.3390/life16091560

**Published:** 2026-09-17

**Authors:** Tongshan Zhu, Shumei Zhang, Mengxin Hao, Ling Wan, Fulei Nie, Guowei Zhang, Bingrui Liu, Chunyu Tian, Xinyue Wang, Haibin Liu, Yijia Li, Yuhan Feng, Di Guo, Nan Li, Yingchao Zhang, Pengcai Liu, Fan Shi, Shuoqian Ma, Rui Cao, Liangdan Sun, Xiaoming Song

**Affiliations:** 1 Hebei Key Laboratory for Chronic Diseases, Tangshan Key Laboratory for Preclinical and Basic Research on Chronic Diseases, School of Basic Medical Sciences, North China University of Science and Technology, Tangshan 063210, China; masterju@stu.ncst.edu.cn (T.Z.); haomengxin@stu.ncst.edu.cn (M.H.); zhangguowei@ncst.edu.cn (G.Z.); a079377@163.com (Y.F.); mashuoqian@ncst.edu.cn (S.M.); 2 School of Life Sciences, North China University of Science and Technology, Tangshan 063210, China; zhangshumei@stu.ncst.edu.cn (S.Z.); drizzling@stu.ncst.edu.cn (L.W.); niefulei@126.com (F.N.); wangxinyue202409@126.com (X.W.); liuhaibinhappy1013@126.com (H.L.); 15127289246@163.com (Y.L.); msguodi@163.com (D.G.); limanxi1989@163.com (N.L.); zhangyc@ncst.edu.cn (Y.Z.); 3 Key Laboratory for Quality of Salt Alkali Resistant TCM of Hebei Administration of TCM, North China University of Science and Technology, Tangshan 063210, China; liupengcai@ncst.edu.cn (P.L.); shifan@ncst.edu.cn (F.S.); 4 Health Science Center, North China University of Science and Technology, Tangshan 063210, China; liubingrui@ncst.edu.cn; 5 Traditional Chinese Medical College, North China University of Science and Technology, Tangshan 063210, China; tianchunyu@ncst.edu.cn

**Keywords:** pancreatic ductal adenocarcinoma (PDAC), cepharanthine (CEP), PD-1/PD-L1 pathway, PI3K/Akt/mTOR pathway

## Abstract

Pancreatic ductal adenocarcinoma (PDAC) is a highly lethal malignancy, and gemcitabine (GEM) resistance is a major contributor to its poor clinical outcomes, generating an urgent need for novel therapeutic strategies targeting tumor proliferation and tumor-cell-intrinsic malignant signaling. This study aimed to investigate the synergistic anti-PDAC efficacy and underlying mechanism of cepharanthine (CEP) combined with GEM. A series of in vitro and in vivo experiments were performed to evaluate the antitumor activity, synergistic effect and preliminary in vivo safety of the CEP-GEM combination. Bioinformatics analysis coupled with experimental validation was applied to dissect the underlying mechanisms. The results demonstrated that CEP produced marked synergistic effects with GEM to suppress PDAC growth both in vitro and in vivo. Mechanistic investigations revealed that CEP blocked the PI3K/AKT/mTOR pathway and reduced tumor-cell-intrinsic PD-1/PD-L1 levels, thereby facilitating tumor-cell apoptosis, restraining cell migration and invasion, and sensitizing GEM-relatively insensitive AsPC-1 cells to GEM. Under our experimental conditions, no obvious histopathological injury or prominent systemic toxicity was observed. In conclusion, this work provides preclinical evidence showing that the CEP-GEM combination exerts anti-tumor activity via dual-pathway suppression in PDAC cells, and supports further translational research for PDAC.

## 1. Introduction

Pancreatic ductal adenocarcinoma (PDAC) is a lethal malignancy with a dismal 5-year survival rate below 10% [1]. Gemcitabine (GEM) historically formed the backbone of systemic therapy, but its efficacy as monotherapy is limited by intrinsic and acquired resistance [2,3]. Contemporary first-line management has shifted toward multi-agent regimens—FOLFIRINOX (or modified FOLFIRINOX), gemcitabine plus nab-paclitaxel [4], and NALIRIFOX [5]—which are preferred for fit patients, whereas GEM monotherapy is largely reserved for those with poor performance status or significant comorbidities [6]. GEM nevertheless remains a core component of several standard regimens, and resistance to GEM-based chemotherapy persists as a major clinical obstacle. Although immune checkpoint inhibitors and targeted agents have been explored as adjuncts, their use is constrained by immune-related adverse events, acquired resistance, and the low immunogenicity of PDAC [7,8]. These limitations underscore an urgent need for novel, tolerable therapeutic strategies.

Natural products, with their inherent structural diversity and multi-target potential, represent a valuable reservoir for anticancer drug discovery. Cepharanthine (CEP), a bisbenzylisoquinoline alkaloid derived from *Stephania cepharantha Hayata*, has demonstrated antiproliferative activity, partially attributed to modulation of the PI3K/Akt/mTOR pathway [9]. However, its clinical development faces one major hurdle: the unclear mechanistic basis for reversing GEM resistance—a promising yet untapped therapeutic strategy in PDAC treatment [10,11].

To address this knowledge gap, we further investigated its combinatorial therapeutic potential with GEM. Herein, we demonstrate that CEP synergizes with GEM to effectively suppress PDAC progression both in vitro and in vivo. Mechanistically, we reveal that CEP mediates dual-pathway blockade: it concurrently inhibits the canonical PI3K/Akt/mTOR proliferative axis and downregulates the tumor-intrinsic immune checkpoint PD-1/PD-L1. Accordingly, this study establishes a low-toxicity combinatorial strategy to enhance the efficacy of GEM in relatively GEM-insensitive PDAC cells, bridging an important translational gap between natural product research and clinically applicable therapies for PDAC.

## 2. Materials and Methods

### 2.1. Cell Lines

The human PDAC cell lines AsPC-1 and MIA PaCa-2 were gifted by the Institute of Medicinal Biotechnology, Peking Union Medical College. Cells were cultured in RPMI 1640 medium supplemented with 10% fetal bovine serum (FBS), penicillin G (100 units/mL), and streptomycin (100 μg/mL), and maintained in a humidified incubator (CellXpert, Eppendorf SE, Hamburg, Germany) at 37 °C with 5% CO_2_. All cells were used within 15 passages.

### 2.2. Animals

Female BALB/c nude mice aged 6–8 weeks (body weight range: 18–22 g) were purchased from Beijing Vital River Laboratory Animal Technology Co., Ltd. (Beijing, China). All animal procedures were performed in strict accordance with the guiding principles outlined in the National Research Council’s *Guide for the Care and Use of Laboratory Animals*. The protocol of animal experiment listed below was reviewed and approved by the Laboratory Animal Ethical and Welfare Committee of North China University of Science and Technology. (Approval No. 2024SY3083).

### 2.3. Drugs and Reagents

Gemcitabine hydrochloride (98% purity) was obtained from Shanghai yuanye Bio-Technology Co., Ltd. (Shanghai, China). CEP was obtained from Shanghai Aladdin Biochemical Technology Co., Ltd. (Shanghai, China). RPMI 1640 medium, phosphate-buffered saline (PBS), trypsin, fetal bovine serum (FBS), and bovine serum albumin (BSA) were purchased from Gibco (Thermo Fisher Scientific, Waltham, MA, USA). The Cell Counting Kit‑8 (CCK‑8) assay reagent was purchased from Vazyme (Vazyme Biotech, Nanjing, China). The cell apoptosis detection kit was purchased from Beyotime (Beyotime Biotechnology Co., Ltd., Haimen, China). Matrix-Gel™ basement membrane matrix (growth-factor-reduced, phenol-red-free, Cat. No. C0376) was obtained from Beyotime Biotechnology (Shanghai, China). The primary antibodies used in this study were as follows: rabbit anti-active and pro-Caspase-3 recombinant monoclonal IgG (1:1000 for WB, #ET1608-64, clone SU38-04; Hangzhou Huaan Biotechnology Co., Ltd., Hangzhou, China); rabbit anti-Bcl-2 polyclonal IgG (1:1500 for WB, #26593-1-AP; Proteintech Group, Inc., Rosemont, IL, USA.); rabbit anti-BAX polyclonal IgG (1:60,000 for WB, #50599-2-Ig; Proteintech Group, Inc., Rosemont, IL, USA); rabbit anti-Phospho-PI3K p85 (Tyr467)/p55 (Tyr199) recombinant monoclonal IgG (1:1000 for WB, #HA721672, clone PSH01-38; Hangzhou Huaan Biotechnology Co., Ltd., Hangzhou, China); rabbit anti-AKT1/2/3 recombinant monoclonal IgG (1:2000 for WB, #HA721870, clone JE75-09; Hangzhou Huaan Biotechnology Co., Ltd., Hangzhou, China); rabbit anti-Phospho-AKT (Ser473) recombinant monoclonal IgG (1:2000 for WB, #HA722129, clone PSH04-44; Hangzhou Huaan Biotechnology Co., Ltd., Hangzhou, China); rabbit anti-mTOR recombinant monoclonal IgG (1:5000 for WB, #ET1608-5, clone SU30-00; Hangzhou Huaan Biotechnology Co., Ltd., Hangzhou, China); mouse anti-Phospho-mTOR (Ser2448) monoclonal IgG1 (1:1000 for WB, #HA600094, clone A5D5; Hangzhou Huaan Biotechnology Co., Ltd., Hangzhou, China); rabbit anti-GAPDH polyclonal IgG (1:3000 for WB, #AF7021; Affinity Biosciences LTD, Liyang, China); rabbit anti-PD-1 monoclonal IgG (1:1000 for WB, #A85455, clone 7A38; Nature Biosciences, Hangzhou, China); rabbit anti-PD-L1 (CD274) monoclonal IgG (1:1000 for WB, #A78758, clone 54Z4; Nature Biosciences, Hangzhou, China). HRP-conjugated goat anti-rabbit IgG (H + L) (1:5000 for WB, #R00001; Nature Biosciences, Hangzhou, China) was used as the secondary antibody.

### 2.4. Preparation of Cepharanthine

For in vitro assays, CEP powder was dissolved in dimethyl sulfoxide (DMSO) to prepare a stock solution, aliquoted, and stored at −80 °C. The stock was diluted with cell culture medium to working concentrations immediately before use, and the final DMSO concentration was maintained at ≤0.1% (*v*/*v*). For in vivo animal administration, the CEP was diluted with sterile phosphate-buffered saline (PBS). Given the poor aqueous solubility of CEP, the mixture was subjected to 37 °C water-bath ultrasonication (GA020C, Shenzhen Guanbo Technology Industrial Co., Ltd., Shenzhen, China).to enhance the dispersion of the drug in the aqueous phase. The solution was freshly prepared immediately prior to each intraperitoneal injection. No additional solubilizers were used in the animal experiments of this study, and visual inspection was performed before each injection to confirm the absence of visible precipitation.

### 2.5. CCK-8 Assay for Cell Viability

AsPC-1 and MIA PaCa-2 cells in logarithmic growth phase were harvested and seeded into 96-well plates at 6000 cells/well in 180 μL of medium. After 12–24 h of incubation (37 °C, 5% CO_2_) to allow attachment, cells were treated with various concentrations of drugs for 24, 48, or 72 h. Control groups received normal medium, and blank groups contained medium without cells. Four to six replicates were used per group. At each time point, 100 μL of 1:10 diluted CCK-8 solution was added to each well and incubated for 0.5–1 h at 37 °C. Optical density (OD) was measured at 450 nm using a microplate reader (EnVision 2105, PerkinElmer, Inc., Waltham, MA, USA). Cell viability was calculated as: Cell viability (%) = [(OD_450_ of experimental group − OD_450_ of blank group)/(OD_450_ of control group − OD_450_ of blank group)] × 100%.

### 2.6. Flow Cytometric Analysis of Apoptosis

Logarithmic-phase AsPC-1 and MIA PaCa-2 cells were seeded in 6-well plates at 4 × 10^4^ cells/mL. After 24 h, cells were treated with 10 μmol/L GEM, 5 μmol/L CEP, or their combination, with untreated cells serving as controls. At 48 h after treatment, culture supernatants containing detached cells were collected, while adherent cells were washed with PBS and trypsinized. The detached and adherent cells were pooled, centrifuged at 1000 *g* for 5 min, resuspended in PBS, and counted. Aliquots of 1 × 10^5^ cells were stained with an Annexin V-FITC Apoptosis Detection Kit (Cat. No. C1062S, Beyotime Biotechnology Co., Ltd., Haimen, China). in the dark at room temperature for 10–20 min according to the manufacturer’s instructions and analyzed using a Beckman Coulter flow cytometer (CytoFLEX LX, Beckman Coulter, Inc., Brea, CA, USA). For data analysis, cellular debris was excluded based on FSC/SSC characteristics, and Annexin V-FITC/PI-positive populations were quantified using quadrant gating. Unstained and single-stained controls were used for fluorescence compensation and gate setting. Cells negative for both Annexin V and PI were considered viable, whereas Annexin V-positive/PI-negative and Annexin V-positive/PI-positive cells were considered early and late apoptotic cells, respectively.

### 2.7. Transwell Analysis and Wound Healing Assay

For migration assays, Transwell inserts were placed in 6-well plates, with serum-free medium added to the upper chambers. The lower chambers contained 10 μmol/L GEM, 5 μmol/L CEP, or their combination (10 μmol/L GEM + 5 μmol/L CEP). Cells were washed, trypsinized, resuspended in serum-free medium, counted, and seeded into the upper chambers. After 48 h, cells migrated to the lower membrane surface were washed with PBS, stained with crystal violet, and imaged. For invasion assays, the same protocol was performed except that insert membranes were pre-coated with Matrigel, with identical drug concentrations in the lower chambers. For wound healing assays, cells were cultured to 90% confluence in 6-well plates. Vertical scratches were made with a sterile 200 μL pipette tip. Following baseline imaging at 0 h, cells were treated with the above-mentioned drug concentrations in serum-free medium to remove serum-derived growth factors and arrest cell proliferation. Microscopic images were captured at 24 h and 48 h thereafter.

### 2.8. Study on ADME, Drug-Likeness and Toxicity Prediction of CEP

ADMETlab version 2.0 was employed to predict its toxicity, including organ toxicity, toxic pathways, and toxicophore rules.

### 2.9. Animal Experiment

In the in vivo animal experiments, therapeutic administration was performed after the establishment of the disease model. Thirty-five female BALB/c nude mice were subcutaneously inoculated with gemcitabine-resistant AsPC-1 PDAC cells (1 × 10^7^ cells/mL, 200 μL per mouse) into the axilla. When tumor volumes reached approximately 100 mm^3^, mice were randomized into groups (*n* = 6 per group) based on tumor size and body weight. (A total of five mice with poor tumor formation efficiency were excluded.) Randomization was performed on day 0. Treatments were administered from day 1 to day 18, and all mice were euthanized on day 19 for tumor excision, weighing and calculation of tumor inhibition rates. The experimental groups were as follows: the Control group received no treatment; the gemcitabine (positive control) group was given 60 mg/kg GEM via intraperitoneal injection on days 1 and 11 (2 doses in total); the cepharanthine group received daily injection of 20 mg/kg CEP from day 1 to day 18; combination group 1 was treated with GEM (60 mg/kg, 2 doses on days 1 and 11) plus 10 mg/kg CEP via daily injection from day 1 to day 18; and combination group 2 was treated with GEM (60 mg/kg, 2 doses on days 1 and 11) plus 20 mg/kg CEP via daily injection from day 1 to day 18. For in vivo administration, both GEM and CEP were delivered to mice via intraperitoneal (i.p.) injection. Both agents were freshly prepared in sterile phosphate-buffered saline (PBS) immediately prior to administration, and water bath sonication was employed to improve the solubility of CEP in PBS.

The exclusion criteria were prospectively predefined before treatment initiation as part of the experimental protocol. Animals were excluded from the final endpoint analysis only when they met one of the predefined criteria, including poor general condition associated with physical injury, unexpected death during the treatment period, or inability to obtain subsequent tumor-volume measurements because the tumor became impalpable. One mouse per group was excluded according to these criteria, resulting in a final sample size of five mice per group for the statistical analysis and result presentation. Tumor diameters and body weights were recorded every 2 days. Tumor volume (V) was calculated as V = ab^2^/2 (a: major axis; b: minor axis). Tumor growth curves and body weight changes were plotted.

### 2.10. Ki67 Immunohistochemical Staining

Paraffin-embedded tumor sections were dewaxed and rehydrated. Endogenous peroxidase was blocked with 3% H_2_O_2_. After antigen retrieval, sections were blocked with 5% BSA, incubated with anti-Ki67 primary antibody at 4 °C overnight, then with secondary antibody for 30 min at room temperature. Signals were developed with DAB, counterstained with hematoxylin, and mounted.

### 2.11. TUNEL Assay

Sections were incubated with TdT + dUTP at 37 °C and counterstained with DAPI (Beyotime Biotechnology, Shanghai, China). The sections were then observed under a microscope (DMi8 automated, Leica Microsystems CMS GmbH, Wetzlar, Germany).

### 2.12. Hematoxylin-Eosin Staining

Mouse organs (heart, liver, spleen, lung, kidney) were fixed in 10% neutral formalin, paraffin-embedded, and sectioned. After dewaxing and rehydration, sections were stained with hematoxylin and eosin sequentially, then dehydrated, cleared, and mounted. Tissue morphology was observed under a light microscope (DMi8 automated, Leica (Leica Microsystems CMS GmbH), Wetzlar, Germany), with images captured.

### 2.13. Network Pharmacology Analysis

Putative protein targets of CEP were retrieved from the SuperPred (https://prediction.charite.de, accessed on 10 December 2025) and Swiss Target Prediction databases. The retrieved target identifiers were converted to official gene symbols using the Universal Protein Resource (UniProt, https://www.uniprot.org, accessed on 10 December 2025) to ensure consistent nomenclature for subsequent analyses. Duplicate target entries were removed, and entries that could not be unambiguously mapped to official gene symbols were excluded. PDAC-associated protein targets were extracted from the GeneCards database (https://www.genecards.org, accessed on 15 December 2025). The resulting PDAC-associated target list was similarly standardized to official gene symbols, with duplicate or nonstandard/unresolvable identifiers removed before downstream analysis. Overlapping targets between CEP and PDAC were identified using Venny 2.1.0 online tool (https://bioinfogp.cnb.csic.es/tools/venny/, accessed on 15 December 2025), and a Venn diagram was generated to visualize shared targets. Overlapping targets between CEP and PDAC were imported into Cytoscape 3.9.1 (https://cytoscape.org, accessed on 17 December 2025) to construct a compound-target-disease network. Subsequently, these overlapping targets were submitted to the STRING database (version 11.5, https://cn.string-db.org, accessed on 18 December 2025) with the species restricted to Homo sapiens and a minimum interaction confidence threshold set to ≥0.7 (highest confidence) to generate the PPI network. The resultant interaction data were then visualized and refined using Cytoscape 3.9.1 for topological analysis. Gene Ontology (GO) functional enrichment and Kyoto Encyclopedia of Genes and Genomes (KEGG) pathway enrichment analyses were conducted on the Microbioinfo online platform. Terms and pathways with adjusted *p*-value (FDR) < 0.05 were defined as significantly enriched entries, and all enrichment outcomes were visualized for subsequent analysis.

### 2.14. Molecular Docking and Visualization

The three-dimensional structure of the small-molecule ligand CEP was downloaded from PubChem (https://pubchem.ncbi.nlm.nih.gov/, accessed on 20 December 2025). Crystal structures of target proteins were retrieved from the RCSB-PDB database with the following PDB IDs: 1ab2, 1h10, 2i8n, 1aue, 1svc, 2enq, 1pks, 8w50. For MTOR (PDB ID: 1aue), chains A and B were retained because the biological functional complex requires both chains. For TOP2A (PDB ID: 8w50), chains A, B, C and D corresponding to its biological tetramer assembly were all kept as the receptor. For the remaining targets, only the biologically relevant single chain was selected, and redundant chains, co-crystallized ligands, and all water molecules were removed using PyMOL (3.1.6.1). Receptor and ligand protonation states were processed at physiological pH 7.4 within AutoDockTools (1.5.7); only polar hydrogens were added to protein receptors, and Gasteiger partial charges were assigned for both receptors and the CEP ligand. Docking-box center coordinates, box dimensions (grid points), and grid spacing (0.375 Å) for each target are summarized in Table 1. Nine independent docking runs were performed for each receptor-ligand pair, and the pose with the lowest binding affinity (kcal/mol) was selected for subsequent structural visualization in PyMOL.

### 2.15. Molecular Dynamics Simulation

All-atom molecular dynamics (MD) simulations of the CEP-PD-L1 complex were performed using GROMACS 2025. The protein was parameterized with the AMBER99SB-ILDN force field. Topology and coordinate files for ligand CEP were generated via the ACPYPE web server (https://bio2byte.be/acpype/, accessed on 12 July 2026) using AM1-BCC partial atomic charges. The complex was solvated in a cubic box with the TIP3P explicit water model, maintaining a minimum distance of 1.0 nm between the protein surface and box boundaries. Na^+^ and Cl^−^ ions were added to neutralize the system and achieve a 0.15 M salt concentration. The system was energy-minimized using the steepest descent algorithm. Two successive 1 ns equilibration steps were conducted under NVT and NPT ensembles. Temperature was maintained at 300 K with the V-rescale thermostat, and pressure was regulated at 1 bar using the Parrinello-Rahman barostat. Three independent 100 ns production simulations (Rep 1, Rep 2, and Rep 3) were conducted using different initial random velocities. A 2 fs integration time step was applied, and coordinates were saved every 10 ps. Binding free energies were calculated using the MM-PBSA method.

### 2.16. Bioinformatic Analysis of Public Clinical Dataset

Gene expression profiles and corresponding clinical follow-up information were retrieved from the Gene Expression Omnibus (GEO) database (GSE28735). The dataset comprised 90 samples, including 45 paired PDAC tumor and adjacent non-tumor tissues from 45 patients, profiled using the Affymetrix Human Gene 1.0 ST microarray platform (GPL6244). The GEO-provided expression matrix consisted of RMA-normalized, gene-level expression values.

For PD-1 and PD-L1 differential expression analysis (the RMA-normalized gene-level expression matrix was analyzed using the limma package in R. Probe-to-gene annotation was performed using the GPL6244 platform annotation file. For genes represented by multiple probes, the mean expression value across the corresponding probes was used for subsequent analysis. The paired tumor–adjacent non-tumor design was accounted for by specifying patient ID as the blocking factor, and the within-patient correlation was estimated using the duplicateCorrelation function. Differential expression between tumor and adjacent non-tumor tissues was assessed using a linear model followed by empirical Bayes moderation. *p* values were adjusted for multiple testing using the Benjamini–Hochberg method.

For PD-L1 survival analysis, tumor samples with available survival time and event information were included. Samples with missing or non-positive survival times or missing event status were excluded. Patients were stratified into high- and low-PD-L1 expression groups according to the median PD-L1 expression level. Overall survival was compared using the log-rank test, and Kaplan–Meier survival curves were generated.

For immune cell infiltration analysis, the CIBERSORT algorithm with the LM22 signature matrix was used to estimate the relative proportions of 22 immune cell subtypes in the 90 tissue samples. The resulting infiltration matrix was processed in Python 3.9 using pandas, and the data were reshaped from wide to long format for visualization. Jittered strip plots were generated using matplotlib and seaborn to facilitate visualization of individual samples.

### 2.17. Western Blot Analysis of Protein Expression

Cells in the logarithmic growth phase were treated with 5 μM GEM, 5 μM CEP, or their combination for 48 h. The concentrations used for the mechanistic experiments were selected based on the 48-h dose–response experiments and the corresponding IC_50_ values, together with preliminary concentration-selection experiments. Cells were then harvested and lysed with RIPA lysis buffer on ice for 30 min. The samples were centrifuged at 12,000 rpm for 20 min at 4 °C, and the supernatant was collected. Protein quantification was performed using the BCA assay. An appropriate amount of the protein solution was mixed with 5× loading buffer and denatured in a boiling water bath for 10 min. After loading, proteins were separated by SDS-PAGE and transferred onto a PVDF membrane. The membrane was blocked with 5% non-fat milk for 2 h and then washed three times with TBST. Subsequently, the PVDF membrane was incubated with primary antibodies overnight at 4 °C. Following TBST washes, the membrane was incubated with fluorescent secondary antibodies for 1 h. After final TBST washes, the membrane was scanned using an imaging system (Tanon‑4200, Shanghai Tanon Science & Technology Co., Ltd., Shanghai, China), and gray value analysis was performed with Fiji‑ImageJ software (2.14.0/1.54f, National Institutes of Health, Bethesda, MD, USA).

### 2.18. siRNA Transfection

Prior to transfection, cells were seeded in 6-well plates and incubated at 37 °C with 5% CO_2_ for 24 h. Transfection was performed using an siRNA transfection reagent kit (Jiangsu Genecefe Biotechnology Co., Ltd., Wuxi, China). An untargeted scrambled siRNA was used as the negative control (NC), and a validated siRNA targeting GAPDH was included as a positive control to assess transfection efficiency. GAPDH siRNA was transfected separately in independent wells solely to verify the transfection system. In all groups used for target protein quantification (Control, NC, and target gene siRNA groups), GAPDH was not targeted, its expression remained stable, and it was used as the loading control for Western blot analysis.

### 2.19. HSA Synergy Analysis

The potential interaction between CEP and GEM was evaluated using the Highest Single Agent (HSA) model. Dose–response data for CEP, GEM, and their combinations were analyzed using the online SynergyFinder platform (https://synergyfinder.org/ accessed on 12 July 2026). Three biologically independent dose–response experiments were performed. For each corresponding drug-concentration combination, the viability values obtained from the three independent experiments were averaged to generate a dose–response matrix for HSA analysis. The experimental data were organized as a dose–response matrix containing the concentrations of each drug and the corresponding response values. The HSA model was used to determine whether the observed effect of the drug combination exceeded the effect of the more effective single agent at the corresponding concentrations. The resulting HSA synergy scores were used to quantify the degree of drug interaction. The *p* value generated by SynergyFinder was calculated at the whole-matrix level based on the distribution of HSA synergy scores across non-monotherapy concentration combinations under the null hypothesis of zero synergy (no interaction).

### 2.20. Statistical Methods

Statistical analyses were performed using GraphPad Prism 8.0. Data are presented as mean ± standard deviation (SD). Normality was assessed using the Shapiro–Wilk test, and homogeneity of variance was assessed using Levene’s test. For normally distributed data with homogeneous variance, one-way analysis of variance (ANOVA) followed by Tukey’s multiple-comparison test was used for multi-group comparisons. For longitudinal measurements of tumor volume and body weight, two-way repeated-measures ANOVA followed by Sidak’s multiple-comparison test was used. Dose–response data were fitted by nonlinear regression using a four-parameter logistic model with variable slope to determine IC_50_ values, with 95% confidence intervals (CIs) reported where applicable. The numbers of independent biological experiments and animals are indicated in the corresponding figure legends. All statistical tests were two-sided, and *p* < 0.05 was considered statistically significant. All data met the required assumptions, and thus parametric tests were applied without the need for alternative non-parametric methods.

## 3. Results

### 3.1. In Vitro Antitumor Activity of Cepharanthine

**Figure 1:**
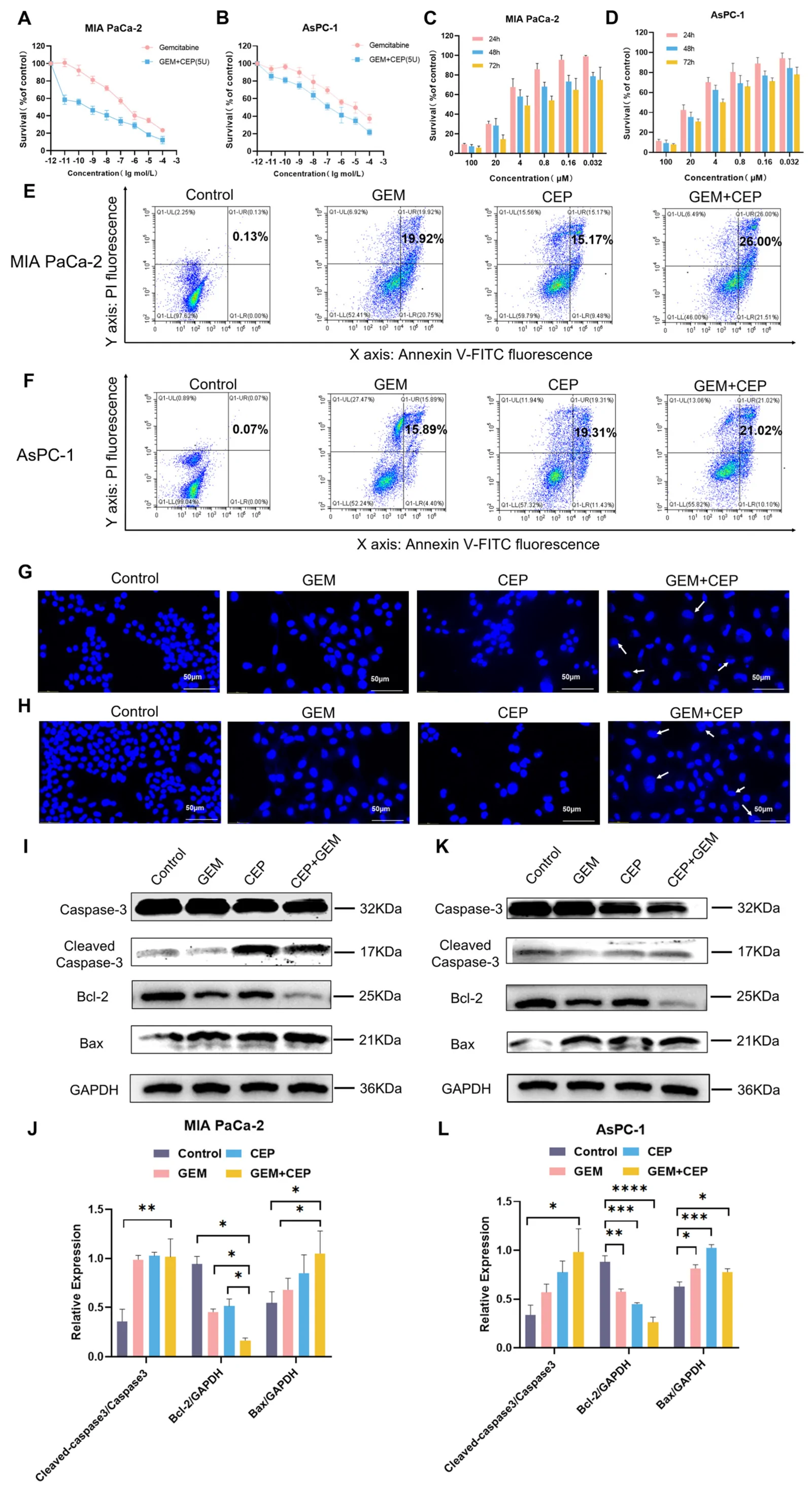
Effects of gemcitabine combined with cepharanthine on viability, apoptosis, and apoptosis-related protein expression in pancreatic cancer cells. (**A**) Concentration-survival curves of MIA PaCa-2 cells treated with GEM alone or in combination with 5 μmol/L CEP at varying concentrations (log mol/L), where the ordinate represents cell viability normalized to the control group (set as 100%). Data are shown as the mean ± SD (*n* = 3 biologically independent experiments), with 4–6 technical replicates per group. (**B**) Concentration-survival curves of AsPC-1 cells treated with gradient concentrations of GEM (log mol/L) alone or combined with 5 μmol/L CEP. Data are shown as the mean ± SD (*n* = 3 biologically independent experiments), with 4–6 technical replicates per group. (**C**) Bar graphs showing the viability of MIA PaCa-2 cells treated with different concentrations of drugs (μM) for 24 h, 48 h, and 72 h. Data are shown as the mean ± SD (*n* = 3 biologically independent experiments), with 4–6 technical replicates per group. (**D**) Bar graphs showing the viability of AsPC-1 cells treated with different concentrations of drugs (μM) for 24 h, 48 h, and 72 h. Data are shown as the mean ± SD (*n* = 3 biologically independent experiments), with 4–6 technical replicates per group. (**E**) Flow cytometry analysis of apoptotic levels in MIA PaCa-2 cells after 48 h of combined drug treatment. (*n* = 3 biologically independent experiments) (**F**) Flow cytometry analysis of apoptotic levels in AsPC-1 cells after 48 h of combined drug treatment. (*n* = 3 biologically independent experiments) (**G**) Morphological changes in apoptotic MIA PaCa-2 cells observed by DAPI staining under fluorescence microscopy (DMi8 automated, Leica Microsystems CMS GmbH, Wetzlar, Germany). (**H**) Morphological changes in apoptotic AsPC-1 cells analyzed by DAPI staining under fluorescence microscopy. (*n* = 3 biologically independent experiments) (**I**) Western blot analysis of Caspase-3, Cleaved Caspase-3, Bcl-2, Bax, and the reference protein GAPDH in MIA PaCa-2 cells from the control group, GEM monotherapy group, CEP monotherapy group, and CEP + GEM combination group. Data are shown as the mean ± SD (*n* = 3 biologically independent experiments). (**J**) Normalized quantitative analysis of the relative expression levels of Cleaved-Caspase 3/Caspase 3, Bcl-2/GAPDH, and Bax/GAPDH with GAPDH as the internal reference. (**K**) Western blot analysis of Caspase-3, Cleaved Caspase-3, Bcl-2, Bax, and the reference protein GAPDH in AsPC-1 cells from the control group, GEM monotherapy group, CEP monotherapy group, and CEP + GEM combination group. Data are shown as the mean ± SD (*n* = 3 biologically independent experiments). (**L**) Normalized quantitative analysis of the relative expression levels of Cleaved-Caspase 3/Caspase 3, Bcl-2/GAPDH, and Bax/GAPDH with GAPDH as the internal reference. A single asterisk (*) signifies a statistically significant difference at the level of *p* < 0.05; double asterisks (**) indicate a statistically highly significant difference with *p* < 0.01; *triple asterisks* (***) denote *p* < 0.001 and quadruple asterisks (****) represent *p* < 0.0001.

CCK-8 assay results showed that in two PDAC cell lines (AsPC-1 and MIA PaCa-2), the half-maximal inhibitory concentration (IC_50_) of CEP alone for cell proliferation exhibited a significant time-dependent decreasing trend. For AsPC-1 cells (Figure 1D), the IC_50_ values of CEP alone at 24 h, 48 h, and 72 h were 12.9, 8.35, and 4.02 μmol/L, respectively. Compared with AsPC-1 cells, MIA PaCa-2 cells were more sensitive to CEP (Figure 1C), with IC_50_ values of 8.55, 6.38, and 2.84 μmol/L at the same time points, all lower than the corresponding values of AsPC-1 cells. When treated with GEM alone, the GEM IC_50_ of AsPC-1 cells was as high as 9 μmol/L (Figure 1A), while that of MIA PaCa-2 cells was only 0.27 μmol/L (Figure 1B). This result further confirmed the relatively lower sensitivity of AsPC-1 cells to GEM. After 48 h of combined treatment with GEM and CEP, cell viability was markedly reduced in both cell lines, and the combination IC_50_ values were 0.13 μmol/L for AsPC-1 and 0.0004 μmol/L for MIA PaCa-2, both much lower than their respective single-agent IC_50_ values.

Flow cytometric analysis (Figure 1E,F) showed that both GEM and CEP monotherapy induced apoptosis in MIA PaCa-2 and AsPC-1 cells, while the GEM + CEP combination group yielded significantly higher total apoptosis rates than either monotherapy group in both cell lines, indicating a consistent synergistic pro-apoptotic effect of the combined regimen against pancreatic ductal adenocarcinoma cells. Morphological observation via DAPI staining (Figure 1G,H) revealed that cells in the control group exhibited regular morphology with uniformly stained nuclei and no obvious apoptotic features. Monotherapy groups presented slight nuclear morphological abnormalities in partial cells, whereas the combination group displayed typical apoptotic phenotypes including nuclear condensation and fragmentation, further corroborating the synergistic apoptosis-inducing effect of the combined treatment.

Subsequently, we detected apoptosis-related proteins. In MIA PaCa-2 cells (Figure 1I,J), compared with the control group, the expression of pro-apoptotic proteins Cleaved-caspase3 and Bax was significantly upregulated, while that of anti-apoptotic protein Bcl-2 was significantly downregulated in CEP, GEM monotherapy and GEM + CEP combination groups. In AsPC-1 cells (Figure 1K,L), the regulatory trend in all treatment groups was consistent. These results indicate that CEP, GEM monotherapy and their combination can regulate the expression of apoptosis-related proteins in both PDAC cell lines, activate pro-apoptotic pathways and inhibit anti-apoptotic pathways.

**Figure 2:**
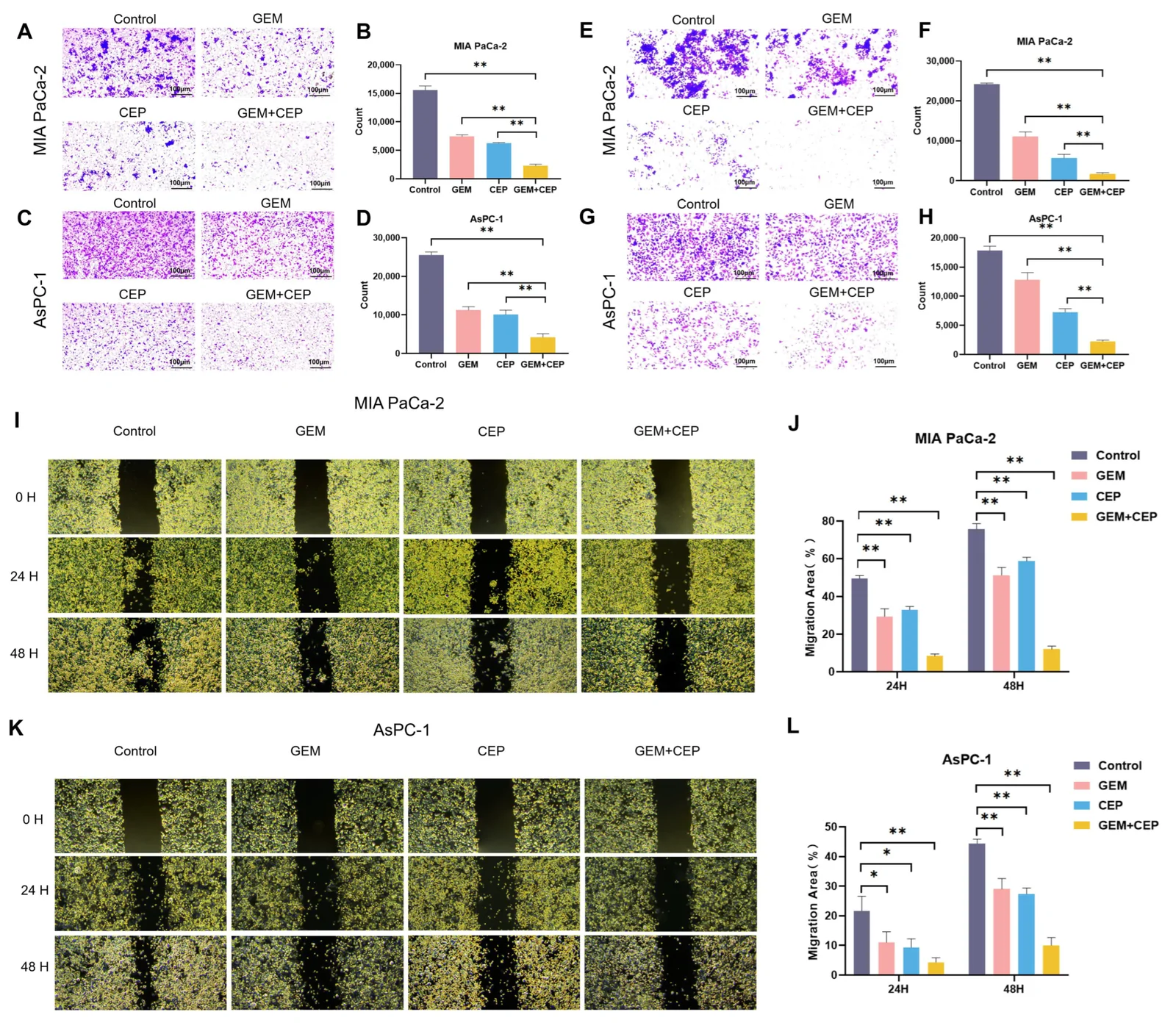
Migration and invasion capacities of MIA PaCa-2 and AsPC-1 cells following control, GEM monotherapy, CEP monotherapy, or GEM + CEP combination therapy. Representative crystal violet-stained images (**A**,**C**,**E**,**G**) and corresponding quantitative analyses of migrated or invaded cell counts (**B**,**D**,**F**,**H**) are presented. (A) MIA PaCa-2 cell migration (*n* = 3). (**B**) quantification of migrated MIA PaCa-2 cells across experimental groups. (**C**) AsPC-1 cell migration under identical treatment conditions (*n* = 3). (**D**) quantification of migrated AsPC-1 cells. (**E**) MIA PaCa-2 cell invasion. (**F**) quantification of invaded MIA PaCa-2 cells. (**G**) AsPC-1 cell invasion following each treatment (*n* = 3). (**H**) quantification of invaded AsPC-1 cells. All images were obtained with crystal violet staining, and the scale bar represents 100 μm for all panels. Double asterisks (**): extremely statistically significant difference (*p* < 0.01). (**I**) MIA PaCa-2 cell wound healing in each group post-treatment (*n* = 3). (**J**) Quantitative analysis of MIA PaCa-2 cell wound healing post-treatment. (**K**) AsPC-1 cell wound healing in each group post-treatment (*n* = 3). (**L**) Quantitative analysis of AsPC-1 cell wound healing post-treatment. Single asterisk (*): statistically significant difference (*p* < 0.05); Double asterisks (**): extremely statistically significant difference (*p* < 0.01). Data are shown as the mean ± SD (*n* = 3 biologically independent experiments).

Then we performed migration and invasion assays. For MIA PaCa-2 cell migration assay, migrated cells were densely distributed in the blank control group. After 48 h of treatment, the number of migrated cells was reduced in the GEM and CEP monotherapy groups, and more significantly in the GEM + CEP combination group (Figure 2A,B). The same trend was observed in AsPC-1 cells, with the number of migrated cells in the combination group significantly lower than that in the monotherapy groups (Figure 2C,D). In the invasion assay, a large number of invasive cells were observed in the blank control group of both cell lines, while extremely few invasive cells were detected in the combination group (Figure 2E–H). To assess the impact of different drug treatments on the horizontal migration of pancreatic cancer cell lines MIA PaCa-2 and AsPC-1, we performed wound healing assays. For MIA PaCa-2 cells, representative images (Figure 2I) and quantification (Figure 2J) showed that at 24 h, migration areas of GEM-, CEP- and GEM + CEP-treated groups were significantly smaller than control (all *p* < 0.01); by 48 h, GEM + CEP co-treatment reduced migration more strongly than single agents (both *p* < 0.01). Consistent results were seen in AsPC-1 cells (Figure 2K,L): all treatments reduced migration vs. control at 24 h, and GEM + CEP co-treatment had stronger effects at 48 h (both *p* < 0.01). Notably, the above migration, invasion and wound-healing assays all quantified the migration and invasion capacity of cells that survived drug treatment. Given the confirmed cytotoxicity of GEM and CEP against PDAC cells, the observed reduction in migrated and invasive cell numbers cannot be exclusively ascribed to specific inhibition of cell motility per se, but may partially stem from drug-induced loss of viable cells. Collectively, these results demonstrate that combined CEP and GEM exerts a stronger suppressive effect on cell migration and invasion in PDAC cells in vitro than single-agent treatment, providing a potential strategy to restrict PDAC migratory and invasive capacity.

### 3.2. In Vivo Toxicity Evaluation and Anti-Tumor Efficacy Verification of Cepharanthine

Prior to carrying out in vivo animal experiments, we utilized the ADMETlab 2.0 platform to conduct systematic in silico toxicity prediction of CEP. The organ-level toxicity probability results revealed prominent potential toxic risks: CEP exhibited high predicted probabilities of hERG inhibition (0.847), human hepatotoxicity (0.619), carcinogenicity (0.868) and respiratory toxicity (0.745). Meanwhile, low risk signals were observed for drug-induced liver injury, eye corrosion and eye irritation. According to toxicophore rule matching outcomes, CEP scored 0 for acute toxicity rule, meaning no acute toxic fragments were matched and there is no obvious acute hazard under conventional exposure conditions. However, its genotoxic-carcinogenicity rule score reached 3, and non-genotoxic carcinogenicity rule score was 1, which together suggested obvious carcinogenic structural alerts. The skin sensitization rule score was 1, while non-biodegradability rule score was 0, indicating weak environmental accumulation potential (Table 1). Notably, all the above computational toxicity predictions only reflect molecular fragment-based structural risk alerts, which cannot be taken as sufficient evidence to verify the in vivo biosafety of CEP. Further systematic animal toxicology experiments are required to accurately evaluate its actual toxic effects.

**Figure 3:**
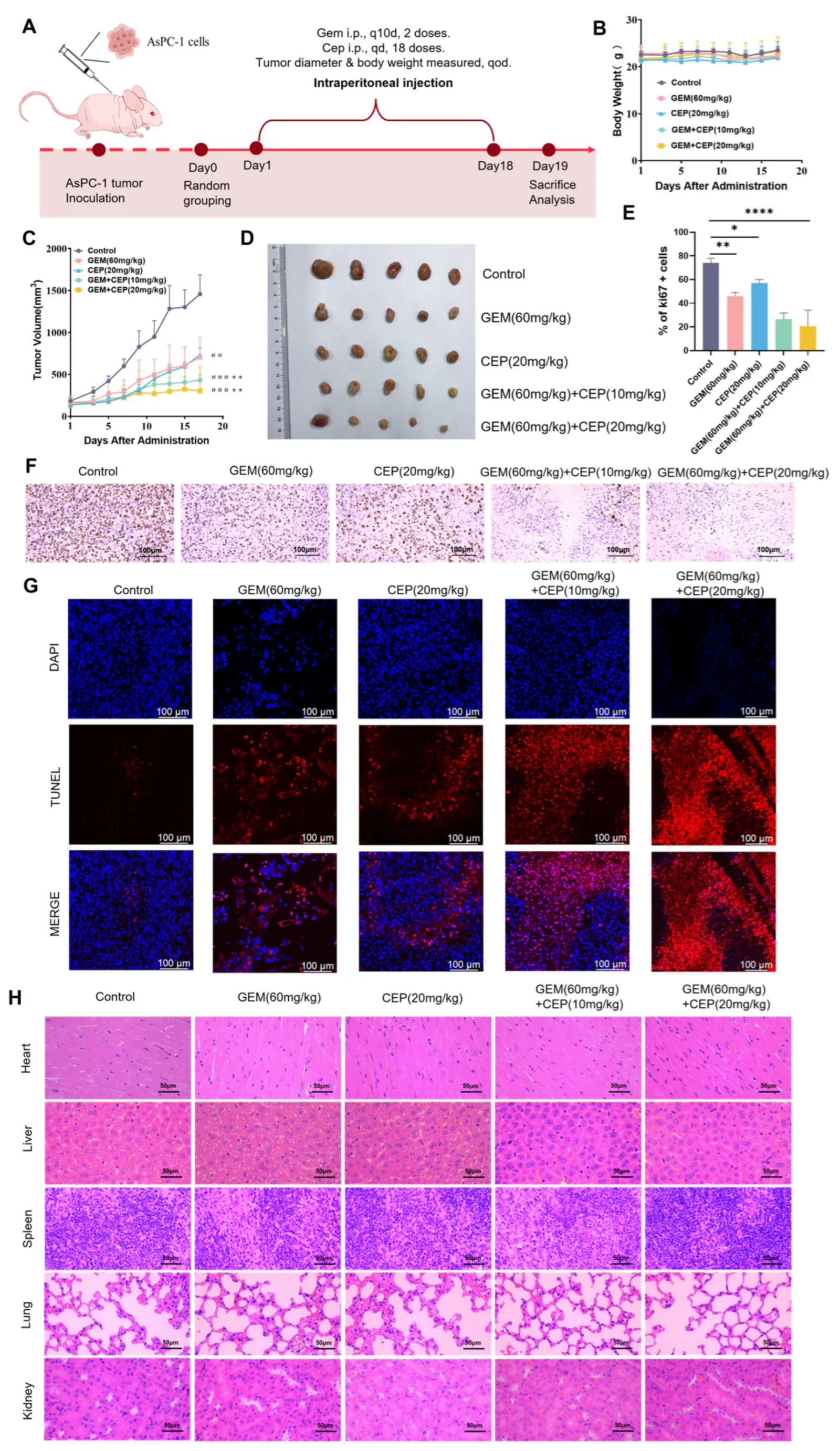
CEP combined with GEM inhibits PDAC growth in AsPC-1 CDX mouse models. (**A**) Experimental schedule. (**B**) Changes in body weight of AsPC-1 CDX model mice treated with drugs. (**C**) Nude mice (*n* = 5) bearing human pancreatic carcinoma AsPC-1 xenograft were treated with GEM (i.p. 60 mg/kg) on Day 1 and Day 11 after tumor formation, respectively. CEP (i.p. 20 mg/kg), GEM + CEP (i.p. 10 mg/kg) or GEM + CEP (i.p. 20 mg/kg) was given once a day. The mean values of tumor volumes in each group are shown with error bars for SD values (*n* = 5). Longitudinal tumor-volume data were analyzed using two-way repeated-measures ANOVA followed by Sidak’s multiple-comparison test. ^##^ represents *p* < 0.05 when compared each group with the control while ^###^, *p* < 0.01. ** indicates *p* < 0.05 compared with the GEM-treated group. (**D**) Images of CEP and GEM in vivo evaluation against AsPC-1 CDX models were taken for each group after sacrifice on Day 19. (**E**,**F**) Sections from paraffin-embedded AsPC-1 xenograft tumors were stained by Ki-67 IHC. Ki-67 data were analyzed using one-way ANOVA followed by Tukey’s multiple-comparison test. Statistical significance: * *p* < 0.05, ** *p* < 0.01, **** *p* < 0.0001. (**G**) Apoptosis in AsPC-1 xenograft tumors was measured using TUNEL assay. (**H**) Representative H&E-stained major organ sections from tumor-bearing mice (scale bar, 50 μm; ×400 magnification).

With reference to the safe dosage range reported in existing studies, we next assessed the in vivo anti-tumor efficacy of CEP combined with GEM in an AsPC-1 cell-derived xenograft (CDX) nude mouse model (Figure 3A) [12,13]. Tumor volume was measured serially throughout the experiment. Tumors grew rapidly in the control group; treatment with single-agent GEM (60 mg/kg) or CEP (20 mg/kg) moderately suppressed tumor growth, whereas combined administration markedly retarded tumor progression, demonstrating synergistic antitumor activity. Body weight was monitored simultaneously (Figure 3B). No obvious body weight loss occurred across all treated groups, indicating favorable safety and low systemic toxicity for monotherapy and combination at the tested doses.

On day 19 post-treatment, nude mice were euthanized and tumor xenografts were surgically excised. Morphological observations revealed that tumor volumes in the combination treatment groups were significantly smaller than those in the control and monotherapy groups, providing direct visual validation of the in vivo anti-tumor efficacy of the combination regimen (Figure 3C,D). Quantitative analyses further confirmed that both GEM and CEP monotherapy exerted significant tumor growth-inhibitory effects. The combined administration of the two agents further augmented the anti-tumor activity in a CEP dose-dependent manner. The therapeutic efficacy of all combination cohorts was significantly superior to that of the control group and the GEM monotherapy group, with all comparisons reaching statistical significance (Table 2). Notably, the GEM + CEP (20 mg/kg) group exhibited relatively high variability in tumor weight. These data should be interpreted as observational findings obtained under the present experimental conditions, rather than definitive evidence of reproducible therapeutic efficacy.

Immunohistochemical IHC staining of proliferation marker Ki-67 displayed intense positive signals in the control group, corresponding to vigorous tumor proliferation. Staining was weakened in monotherapy groups and barely detectable in the combination group (Figure 3E,F), revealing synergistic suppression of tumor proliferation upon combined treatment. TUNEL-DAPI co-staining further exhibited markedly more TUNEL-positive apoptotic cells (red fluorescence) in the combination group versus control and single-drug groups (Figure 3G), confirming strengthened pro-apoptotic efficacy of the combined regimen.

Subsequently, hematoxylin and eosin (H&E) staining was applied to evaluate organ toxicity in tumor-bearing nude mice (Figure 3H). Histopathological observation showed intact morphological structures of heart, liver, spleen, lung and kidney in all treated groups: regular cardiac layers, well-organized hepatic lobules, intact splenic corpuscles, no pulmonary inflammation/edema/necrosis, and undamaged renal glomeruli. No obvious histopathological abnormalities were observed in the major organs between the treatment and control groups, suggesting that no significant signs of systemic toxicity were detected under the experimental conditions employed.

In conclusion, under the experimental conditions tested, CEP combined with GEM markedly suppresses the growth of AsPC-1 xenograft tumors via inhibition of cell proliferation and induction of apoptosis, with no overt toxicity observed. This combination represents a promising potential therapeutic strategy for PDAC.

### 3.3. Analysis of Action Targets and Pathways Based on Network Pharmacology and Molecular Docking

To explore the mechanism by which CEP exerts its anti-pancreatic ductal adenocarcinoma effects, we performed a series of bioinformatic analyses followed by experimental validation.

**Figure 4:**
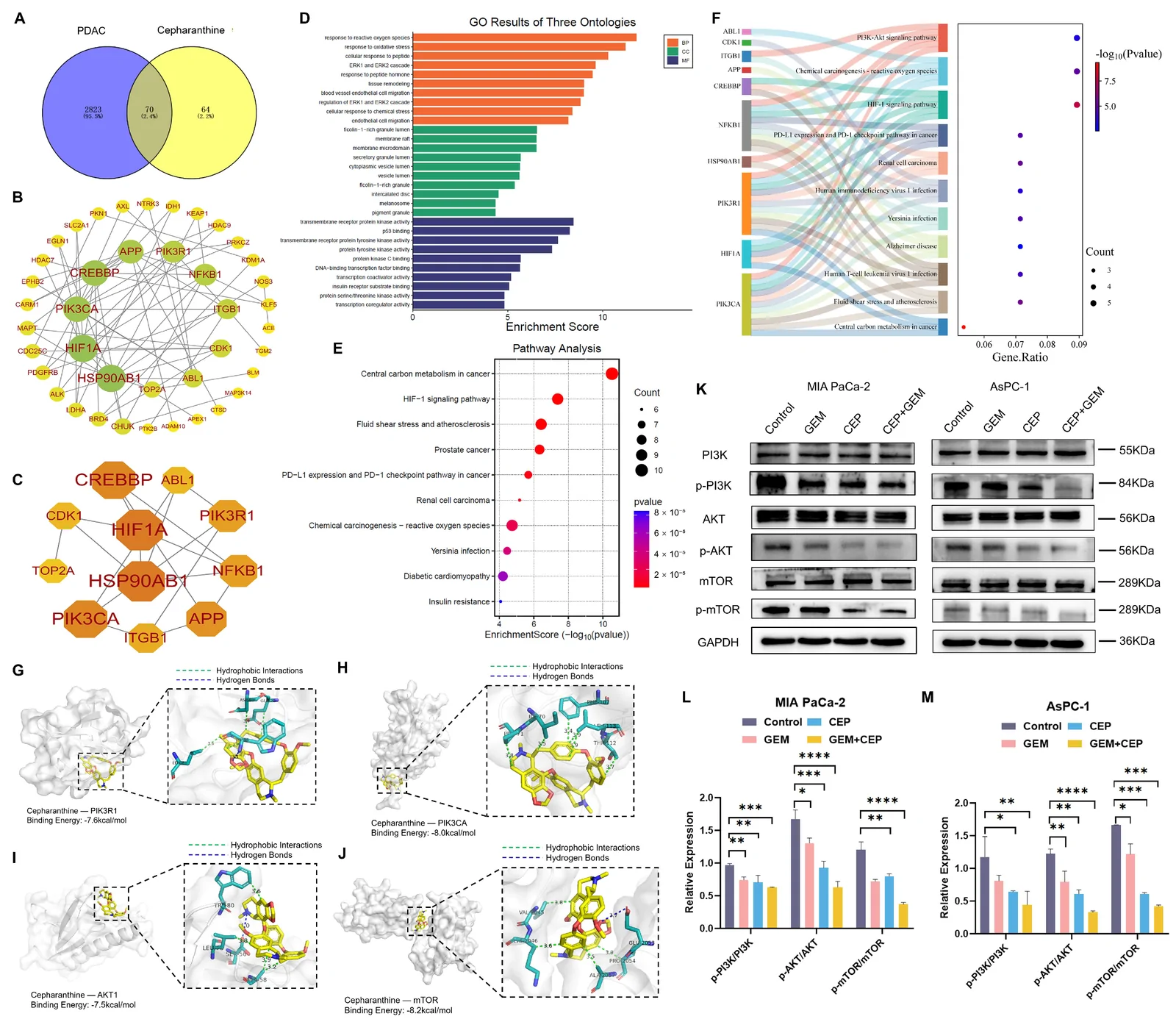
Integrative network pharmacology and experimental validation of CEP targeting the PI3K/AKT/mTOR pathway in PDAC. (**A**) Venn diagram of intersecting genes between PDAC and CEP. (**B**) Protein–protein interaction (PPI) network of intersecting targets for CEP treatment of PDAC. (**C**) PPI network of 11 core intersecting targets. (**D**) GO enrichment analysis of key targets for CEP treatment of PDAC. (**E**) KEGG enrichment analysis of key targets for CEP treatment of PDAC. (**F**) Sankey bubble plot of the top 11 enrichment pathways contained core genes. (**G**–**J**) show the docking configurations of CEP with PIK3R1, PIK3CA, AKT1 and mTOR, respectively. In each subfigure, the left panel depicts the overall structure of the protein, while the enlarged right panel highlights CEP (colored yellow) and the key interaction regions with the protein. Hydrophobic interactions are denoted by green dashed lines, hydrogen bonds by blue dashed lines, and binding energy values are indicated to quantify binding affinity. (**K**) Western blot of PI3K/p-PI3K, AKT/p-AKT, mTOR/p-mTOR in MIA PaCa-2/AsPC-1 cells (GAPDH: internal reference; molecular weights indicated). Data are shown as the mean ± SD (*n* = 3 biologically independent experiments). (**L**,**M**) Quantitative analysis of phosphorylation ratios (p-PI3K/PI3K, p-AKT/AKT, p-mTOR/mTOR) in MIA PaCa-2 (**L**) and AsPC-1 (**M**) cells. Statistics: one-way ANOVA; * *p* < 0.05, ** *p* < 0.01, *** *p* < 0.01, **** *p* < 0.01.

134 target genes of CEP and 2893 PDAC-related genes were screened, yielding 70 overlapping targets via intersection analysis (Figure 4A). These overlapping targets were submitted to the STRING database (limited to Homo sapiens) to obtain protein–protein interaction (PPI) data. Following visualization analysis (Figure 4B,C), core genes including HSP90AB1, HIF1A, PIK3CA, and CREBBP were identified, indicating their pivotal roles in the therapeutic action of CEP against PDAC.

Subsequently, GO functional enrichment analysis of the 70 overlapping targets yielded 1433 entries. The top 10 terms from each category were visualized in a bar chart (Figure 4D). KEGG pathway enrichment analysis identified 102 pathways; the top 10 enriched pathways, such as central carbon metabolism in cancer, HIF-1 signaling pathway, and PD-L1 expression and PD-1 checkpoint pathway in cancer, were presented as a bubble chart (Figure 4E). The top 11 enriched pathways containing core genes were selected by count ranking and further visualized via a Sankey bubble chart (Figure 4F). PIK3R1 and PIK3CA were involved in the largest number of enriched functional terms (e.g., the PI3K-Akt signaling pathway), followed by NFKB3 and HIF1A.

Next, molecular docking and visualization analysis were subsequently performed. Docking results showed that the binding energies of CEP to most core targets were negative. Notably, the absolute binding energies of CEP with PIK3R1, PIK3CA, AKT1, and mTOR all reached ≥ 7.5 kcal/mol. These simulations predicted potential interactions between CEP and the aforementioned proteins, with favorable docking poses and relatively stable complex conformations observed during the simulations. (Figure 4G–J). These findings offer insights into the putative binding modes and affinities between CEP and tumor-associated signaling proteins, providing a structural-biological basis for further exploration of its pharmacological activity and therapeutic potential.

Finally, Western blot analysis was performed to examine the regulatory effects of GEM monotherapy, CEP monotherapy, and their combination on the PI3K/AKT/mTOR signaling pathway. The results demonstrated that the combined treatment significantly downregulated the relative expression levels of p-PI3K, p-AKT, and p-mTOR in both cell lines, with all differences showing statistical significance (Figure 4K–M).

### 3.4. Validation of PD-1/PD-L1 Pathway as a Target of Cepharanthine and Related Mechanism Analysis

**Figure 5:**
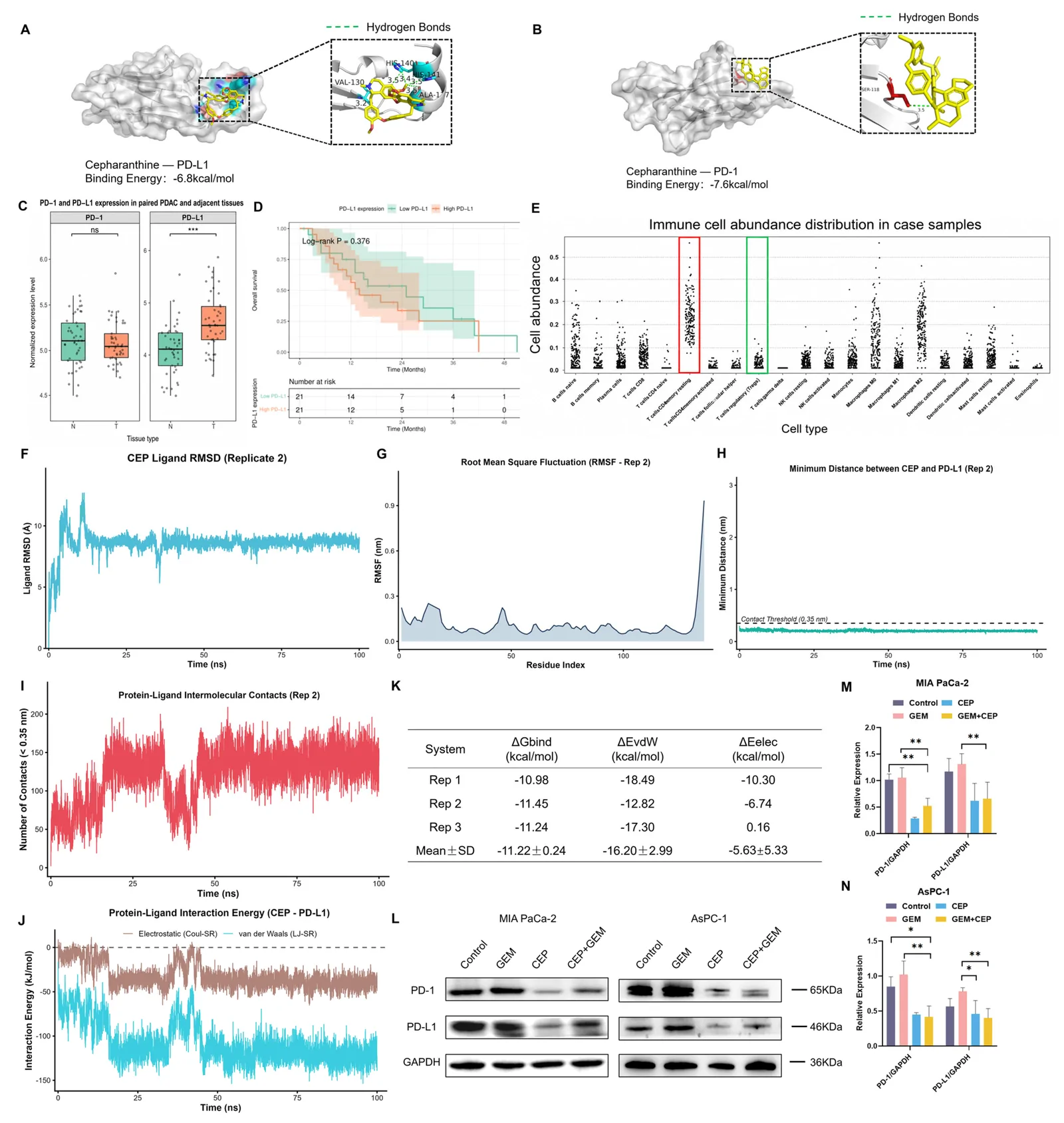
Validation of PD-1/PD-L1 Pathway as a Target of CEP and Related Mechanism Analysis. (**A**,**B**) Show the docking configurations of CEP with PD-1 and PD-L1, respectively. In each subfigure, the left panel depicts the overall structure of the protein, while the enlarged right panel highlights CEP (colored yellow) and the key interaction regions with the protein. Hydrophobic interactions are denoted by green dashed lines, hydrogen bonds by blue dashed lines, and binding energy values are indicated to quantify binding affinity. (**C**) Expression difference in PD-L1 gene between pancreatic ductal adenocarcinoma tissues and adjacent normal tissues. (**D**) Kaplan–Meier survival curve analysis of overall survival difference in pancreatic ductal adenocarcinoma patients stratified into high and low PD-L1 expression groups by median expression level. (**E**) Relative abundance distribution characteristics of various immune cell subsets in pancreatic ductal adenocarcinoma clinical samples. (**F**) Ligand root-mean-square deviation (RMSD). (**G**) Protein root-mean-square fluctuation (RMSF) per residue. (**H**) Minimum distance between the CEP ligand and the PD-L1 binding cleft. (**I**) Number of protein-ligand intermolecular contacts (<0.35 nm). (**J**) Electrostatic and van der Waals interaction energy components. (**K**) Binding free energy and its components for the CEP-PD-L1 complex across three independent MD replicates. (**L**) Western blot analysis of PD-1, PD-L1 and the reference protein GAPDH in cells from the control group, GEM monotherapy group, CEP monotherapy group, and CEP + GEM combination group. Data are shown as the mean ± SD (*n* = 3 biologically independent experiments). (**M**,**N**) Normalized quantitative analysis of the relative expression levels of PD-1/GAPDH and PD-L1/GAPDH with GAPDH as the internal reference. A single asterisk (*) signifies a statistically significant difference at the level of *p* < 0.05; double asterisks (**) indicate a statistically highly significant difference with *p* < 0.01.

Our prior KEGG enrichment analysis also identified the PD-1/PD-L1 pathway. As a core immune checkpoint pathway mediating immune tolerance and tumor immune evasion, this finding suggests that it may serve as a key target for the biological regulatory effects of CEP. Subsequent molecular docking simulations between CEP and PD-1, as well as CEP and PD-L1, demonstrated that the absolute binding energies of CEP to PD-1 and PD-L1 were both ≥ 6.8 kcal/mol (Figure 5A,B), suggesting that CEP may establish relatively stable and favorable-affinity interactions with these two target proteins.

To investigate the expression characteristics and clinical relevance of PD-L1 in PDAC, differential expression and survival analyses were performed using the GSE28735 dataset from the Gene Expression Omnibus (GEO) database. Differential expression analysis was performed using the limma package, accounting for the paired tumor and adjacent non-tumor tissues. PD-L1 expression was significantly higher in PDAC tumor tissues than in paired adjacent non-tumor tissues (log_2_FC = 0.53, FDR = 5.93 × 10^−6^), whereas PD-1 expression showed no significant difference (FDR = 0.638) (Figure 5C). Survival analysis was performed in patients with available survival information, with the median PD-L1 expression used as the cutoff. No statistically significant difference in overall survival was observed between the PD-L1-high and PD-L1-low groups (log-rank *p* = 0.376) (Figure 5D).

To further characterize the immune-cell composition of the clinical samples, CIBERSORT was used to estimate the relative abundance of immune-cell populations from bulk gene-expression profiles (Figure 5E). The estimated abundance of several immune-cell populations varied substantially among individual samples, with relatively broad variation in resting memory CD4^+^ T cells and generally lower estimated levels of Tregs. These findings revealed differences in the estimated immune-cell composition among the analyzed PDAC samples. These differences may reflect heterogeneity in the tumor immune microenvironment and provide a basis for further investigation of potential immunosuppressive mechanisms.

Subsequently, we performed additional molecular dynamics simulations over a 100 ns trajectory to systematically evaluate the dynamic interactions between CEP and PD-L1 (Figure 5F–J). Data for Replicate 1 and Replicate 3 are provided in Supplementary Figures S1 and S2. The ligand root-mean-square deviation (RMSD) plot (Figure 5F) displays values stabilizing in a range of 8 to 10 Å after the initial 15 ns. The Root Mean Square Fluctuation (RMSF) analysis (Figure 5G) measures values below 0.3 nm for the core protein residues. RMSF values increase at the C-terminus. The minimum distance trajectory involving the CEP ligand spacing the PD-L1 pocket (Figure 5H) measures below the 0.35 nm contact threshold across the entire 100 ns period. The intermolecular contact numbers (Figure 5I) record values spanning 100 to 150 interactions at the binding site. The interaction energy profile (Figure 5J) shows van der Waals (vdW) energies ranging from −100 to −150 kJ/mol. Electrostatic energies range from −25 to −50 kJ/mol. The MM-PBSA method calculated binding free energies for the three replicates (Figure 5K). These quantitative results indicate that the CEP ligand maintains a stable binding conformation within the PD-L1 pocket, primarily driven by van der Waals interactions.

To clarify the regulatory role and underlying molecular mechanism of CEP on the PD-1/PD-L1 signaling pathway in pancreatic ductal adenocarcinoma, we performed Western blot assays. The results showed that compared with the positive drug GEM group, both the CEP monotherapy group and the CEP-GEM combination therapy group significantly downregulated the protein expression levels of PD-1 and PD-L1, with all observed differences being statistically significant (Figure 5L–N).

### 3.5. Synergistic Anti-Tumor Effect of CEP Combined with GEM and PD-L1 Regulation Validation

**Figure 6:**
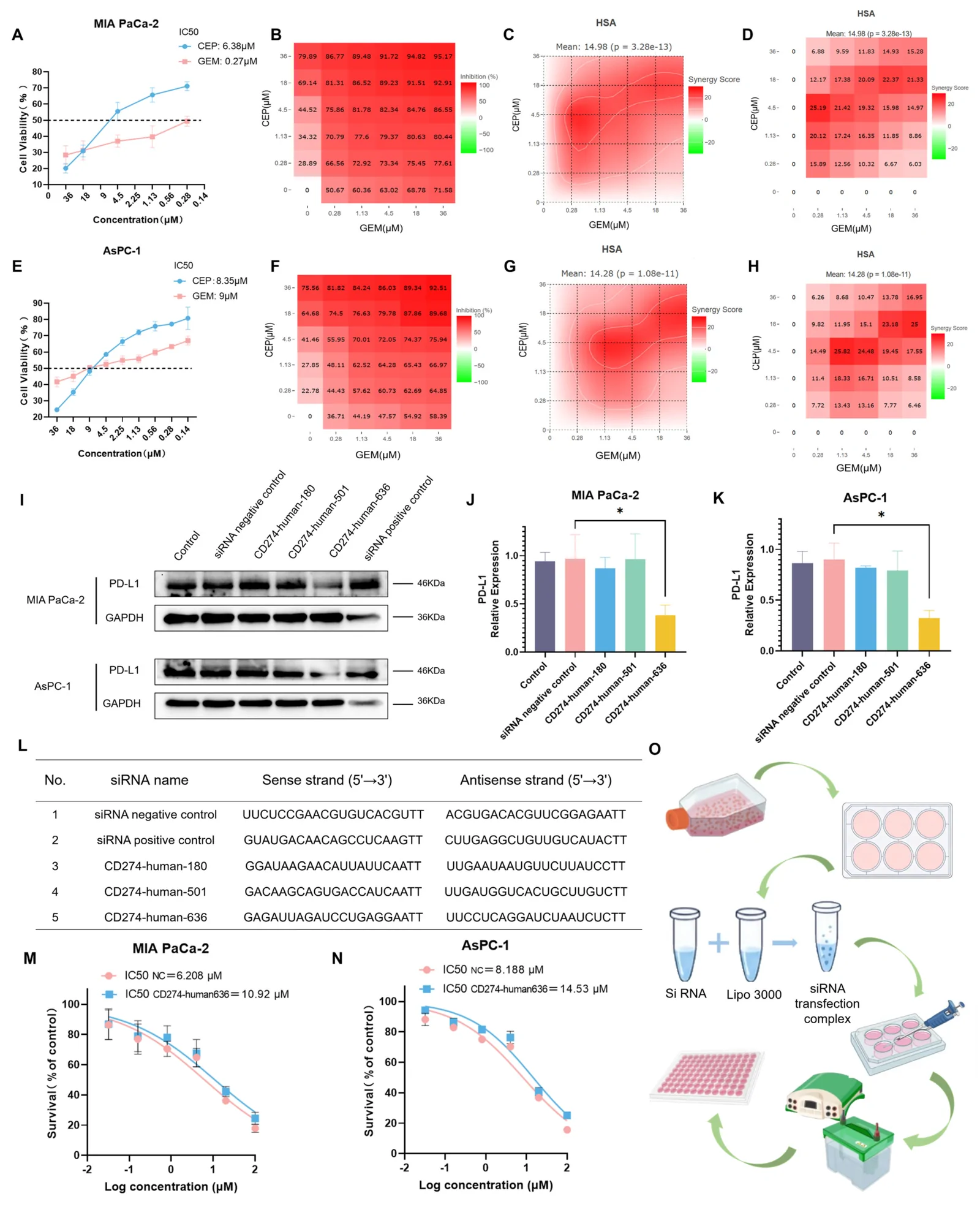
Synergistic anti-tumor effect of CEP combined with GEM and PD-L1 regulation validation. (**A**) Cell viability of MIA PaCa-2 cells following 48 h treatment with CEP or GEM alone. Data are shown as the mean ± SD (*n* = 3 biologically independent experiments), with 4–6 technical replicates per group. (**B**) Proliferation inhibition rate of MIA PaCa-2 cells following 48 h combined treatment with CEP and GEM. Data are shown as the mean ± SD (*n* = 3 biologically independent experiments), with 4–6 technical replicates per group. (**C**,**D**) Synergy heatmap of GEM combined with CEP in MIA PaCa-2 cells analyzed by the HSA model. (**E**) Cell viability of AsPC-1 cells following 48 h treatment with CEP or GEM alone. (**F**) Proliferation inhibition rate of AsPC-1 cells following 48 h combined treatment with CEP and GEM. (**G**,**H**) Synergy heatmap of GEM combined with CEP in AsPC-1 cells analyzed by the HSA model. (**I**) Protein expression levels after siRNA transfection. Data are shown as the mean ± SD (*n* = 3 biologically independent experiments). (**J**,**K**) Quantitative gray-scale analysis of protein expression levels after siRNA transfection. (**L**) Sequences of siRNA oligonucleotides. (**M**) Cell viability curves of PD-L1-knockdown MIA PaCa-2 cells after 48 h CEP treatment. Data are shown as the mean ± SD (*n* = 3 biologically independent experiments), with 4–6 technical replicates per group. (**N**) Cell viability curves of PD-L1-knockdown AsPC-1 cells after 48 h CEP treatment. Data are shown as the mean ± SD (*n* = 3 biologically independent experiments), with 4–6 technical replicates per group. (**O**) Schematic diagram of the siRNA transfection and validation workflow. A single asterisk (*) signifies a statistically significant difference at the level of *p* < 0.05;

To evaluate the combined antitumor effects of CEP and GEM in PDAC cells, a series of in vitro experiments were conducted. Cell viability of two PDAC cell lines was measured after 48 h of treatment with CEP or GEM alone (Figure 6A,E) or in combination (Figure 6B,F). Synergy analysis based on the HSA model revealed a synergistic effect between CEP and GEM, with the strongest synergy observed in the low-to-moderate concentration range (Figure 6C,D,G,H). These findings demonstrate that CEP and GEM exert a synergistic inhibitory effect on PDAC cell viability, particularly at low-to-moderate concentrations.

Subsequently, PD-L1 was knocked down using siRNA transfection. The knockdown efficiencies of the three siRNA sequences, CD274-human-180, CD274-human-501, and CD274-human-636, were 13.7%, 20.0%; 3.81%, 23.1%; and 63.6%, 75.6% in MIA PaCa-2 and AsPC-1 cells, respectively, as verified by Western blotting. Therefore, CD274-human-636, which showed the highest knockdown efficiency in both cell lines, was selected for subsequent functional validation (Figure 6I–K). PD-L1 knockdown significantly attenuated the growth-inhibitory effect of CEP and increased its IC_50_ values in both PDAC cell lines (Figure 6M,N). Specifically, the IC_50_ of CEP increased from 6.208 μM (95% CI, 3.745–10.46 μM) to 10.92 μM (95% CI, 6.341–19.86 μM) in MIA PaCa-2 cells and from 8.188 μM (95% CI, 5.634–11.93 μM) to 14.53 μM (95% CI, 10.69–20.01 μM) in AsPC-1 cells following PD-L1 knockdown. These results indicate that PD-L1 depletion reduces the sensitivity of PDAC cells to CEP, suggesting that PD-L1-related mechanisms are involved in the antitumor activity of CEP and providing a basis for further mechanistic investigation.

## 4. Discussion

This study conducted a series of investigations on CEP following a systematic framework of “pharmacological exploration-mechanism elucidation”. It clarified the synergistic mechanism of CEP in combination with GEM against PDAC. The main scientific contribution of this study lies in the exploration of concurrent modulation of two key signaling axes, providing new academic support for the mitigation of chemoresistance in PDAC.

Despite the shift toward multi-agent first-line regimens, PDAC remains highly lethal, and resistance to GEM-based chemotherapy continues to constrain clinical outcomes [14,15]. “Multi-target combination therapy” has thus emerged as a key research direction in this field [16,17]. This study confirmed that the combination of CEP and GEM exerts potent tumor-suppressive effects both in vitro (AsPC-1 and MIA PaCa-2 cell lines) and in vivo (AsPC-1 xenograft model), with the highest tumor inhibition rate reaching 76.3% in the combination group—significantly superior to monotherapy. This finding supports the efficacy of the combination therapy and suggests that CEP may have potential as a natural product-derived synergistic agent. In contrast to synthetic drugs, CEP possesses pleiotropic effects including antiproliferation, pro-apoptosis, and immunomodulation [18]. Notably, no pathological damage to major organs such as the heart, liver, spleen, lungs, and kidneys was observed in in vivo experiments, presenting a novel candidate regimen for the treatment of chemoresistant PDAC.

The mechanistic findings of this study indicate that CEP regulates programmed death ligand 1 (PD-L1) expression in PDAC, providing further insights into its potential role in PDAC combination therapy. As a core immune checkpoint molecule, high PD-L1 expression on tumor cells is a key mediator of immune escape and is closely associated with poor prognosis in PDAC patients—clinically, patients with high PD-L1 expression exhibit poor treatment response and prolonged tumor progression time [17,19,20]. More importantly, chemotherapeutic agents such as GEM can upregulate PD-L1 expression through feedback activation pathways, forming a vicious cycle of “chemotherapy-induced immunosuppression” that severely limits chemotherapeutic efficacy [21]. Therefore, combining chemotherapy with PD-L1-downregulating agents to break this cycle has become a research hotspot in clinical combination therapy for PDAC [22]. Beyond PD-L1, the pro-tumor role of programmed death 1 (PD-1) in PDAC cannot be ignored [23]. Unlike its classical immunosuppressive function on immune cells, PD-1 is abnormally highly expressed in PDAC tumor cells, enhancing tumor cell proliferation and invasion by activating downstream pathways such as MET and Hippo [24,25]. Its high expression is closely linked to shortened overall survival in patients, making it an important oncogene in PDAC [26]. The present findings suggest that the antitumor effects of CEP against PDAC are mediated primarily through direct actions on tumor cells, as evidenced by reduced cell viability and enhanced apoptosis. In addition, CEP treatment decreased the protein expression of PD-L1 and PD-1 in PDAC cells, suggesting that tumor-cell-intrinsic PD-1/PD-L1-related signaling may be involved in the cellular response to CEP. This interpretation is consistent with previous studies showing that, in addition to its canonical role in immune cells, PD-1 can also be expressed intrinsically by pancreatic cancer cells and contribute to the regulation of tumor-cell proliferation and disease progression. However, because the present study employed a tumor-cell monoculture system in the absence of immune cells, the observed changes in PD-1 and PD-L1 expression should be interpreted primarily as alterations in tumor-cell-intrinsic signaling rather than as evidence of functional modulation of the canonical PD-1/PD-L1 immune checkpoint. Overall, these findings suggest a potential association between CEP treatment and tumor-cell-associated regulation of PD-1/PD-L1, while the functional significance of this regulation in tumor–immune cell interactions and antitumor immunity remain to be established.

**Figure 7:**
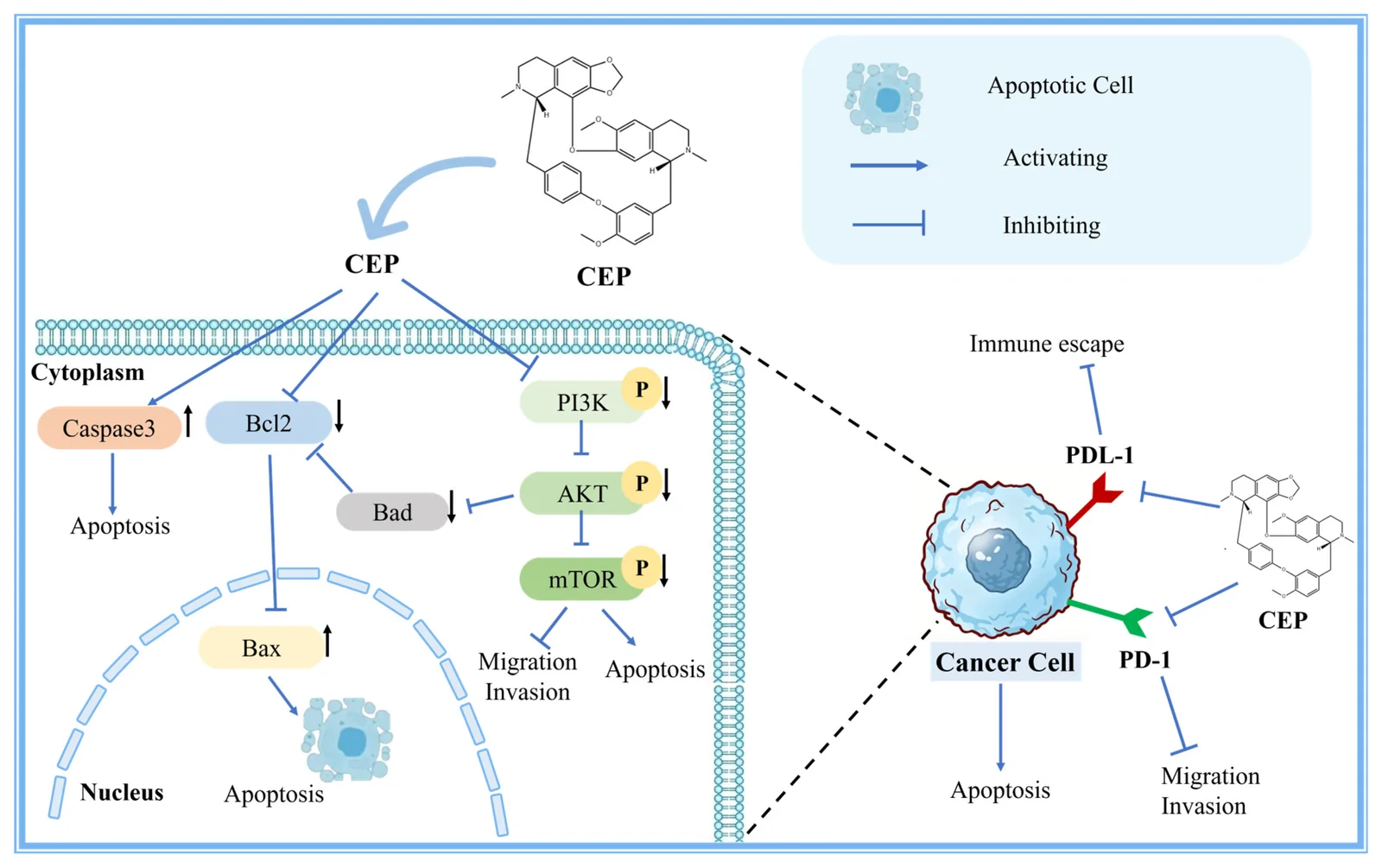
Mechanistic pathway diagram of the action of CEP against PDAC.

Furthermore, a mechanistic finding of this study is that CEP was associated with concurrent suppression of the PI3K/AKT/mTOR signaling pathway and modulation of PD-1- and PD-L1-related markers in PDAC cells. In addition, PD-1 and PD-L1 serve as the core proteins of the PD-1/PD-L1 signaling pathway. Building on these findings, we propose a hypothetical model of crosstalk between these two pathways, and put forward HIF-1α/STAT3 as a candidate shared upstream regulatory mechanism underlying this interplay. The PI3K/AKT/mTOR pathway serves as a core axis regulating tumor proliferation, metabolism, and metastasis, and its aberrant activation is a key driver of PDAC initiation, progression, and chemoresistance [27,28]. Meanwhile, immune escape mediated by the PD-1/PD-L1 pathway and chemotherapy-induced PD-L1 upregulation are important causes of poor therapeutic efficacy [29,30]. Collectively, results from molecular docking, molecular dynamics simulations and Western blotting support the finding that CEP can specifically interact with key regulatory proteins such as PI3K (PIK3CA), PD-1 and PD-L1. Drawing on existing literature reporting HIF-1α and STAT3 as common upstream regulators of both pathways, we hypothesize that CEP may exert simultaneous inhibition of both pathways by targeting the HIF-1α/STAT3 axis [31,32]. This logic not only explains the advantage of CEP as a single-agent dual-targeting agent but is also supported by the experimental findings that CEP downregulates the expression of p-PI3K, p-AKT, PD-1, and PD-L1 simultaneously (Figure 7).

Compared with previously reported combinatorial strategies for PDAC, the CEP-GEM combination evaluated in this study exhibits a unique pharmacological profile. While GEM + PD-1 inhibitors integrate chemotherapy and immune checkpoint blockade, off-target effects lead to a high incidence of immune-related adverse events (irAEs), and some patients show no response due to insufficient tumor immunogenicity [33,34]. In contrast, CEP’s regulation of PD-1/PD-L1 is tumor cell-targeted, avoiding the risk of irAEs associated with generalized immunosuppression. GEM + PI3K inhibitors improve efficacy by dual proliferation pathway inhibition, but synthetic PI3K inhibitors are prone to severe adverse reactions such as hyperglycemia and gastrointestinal disorders, and single-pathway targeting easily leads to rapid drug resistance [35,36]. As a naturally derived bioactive compound, CEP exhibits a favorable safety profile and suppresses tumor cell proliferation by modulating two key signaling pathways. As previously reported, its potential immunomodulatory property may delay the onset of chemoresistance [37]. Importantly, the CEP-GEM combination tested in our study markedly enhanced tumor growth inhibition with no detectable pathological injury to major organs, which provides a solid pre-clinical basis for future translational research.

Despite these preliminary findings, the present study has several notable limitations, and some questions remain to be addressed. First, the in vitro functional assays were conducted in only two PDAC cell lines, AsPC-1 and MIA PaCa-2, and were not validated using primary clinical specimens. Therefore, whether the findings observed in the present study are broadly applicable to other PDAC models and clinical settings remains to be determined. Second, all migration, invasion, and wound-healing assays were performed under sustained drug exposure and therefore reflected the migratory and invasive capacities of cells that survived under drug treatment pressure. Because CEP and GEM exert cytotoxic effects on PDAC cells, the observed inhibition of migration and invasion cannot be completely separated from the reduction in viable cell numbers induced by drug treatment and therefore cannot be attributed solely to specific effects on cellular motility. Third, pharmacokinetic analyses and long-term toxicity assessments of CEP were not performed in this study. Consequently, its intratumoral accumulation, metabolic behavior, and long-term safety profile remain insufficiently characterized. In light of these limitations, future studies should focus on two complementary directions. First, the present findings should be validated in a broader range of PDAC models, including an expanded panel of cell lines and, where feasible, primary clinical specimens. Experimental approaches should also be further optimized to minimize the confounding effects of drug-induced reductions in viable cell numbers on the interpretation of migration and invasion assays. In parallel, pharmacokinetic studies and more comprehensive toxicity evaluations will be important for further characterizing the in vivo exposure and safety profiles of CEP. Second, further mechanistic investigations are warranted to elucidate the molecular determinants underlying CEP-associated regulation of PD-L1 and cellular responses to CEP. Genome-wide CRISPR-based screening, combined with targeted genetic and pharmacological validation, may help identify key regulatory factors and provide more definitive experimental evidence for the mechanisms underlying the effects observed in this study.

In summary, by integrating multidisciplinary experimental approaches, this study delineates the concurrent dual-pathway mechanism of CEP combined with GEM against PDAC. The key novelty lies in the dual effects of modulating PD-1/PD-L1 and suppressing the PI3K signaling cascade, which delivers pre-clinical evidence for the anti-tumor efficacy of the CEP-GEM combination in PDAC. These findings advance our knowledge of the anti-tumor properties of compounds derived from traditional Chinese medicine and encourage further exploration of natural products for multi-target combination regimens. Both its methodological approach and mechanistic findings provide valuable insights for further research and may have potential implications for future translational investigation.

## Figures and Tables

**Table 1:** Organ level toxicity profile of Cepharanthine.

Sr. No	Toxicity Parameter	Inference
1.	hERG blockers	0.847
2.	Human Hepatotoxicity	0.619
3.	DILI (Drug induced liver injury)	0.022
4.	Skin sensitization	0.584
5.	Carcinogenicity	0.868
6.	Eye–corrosion	0.0
7.	Eye–irritation	0.007
8.	Respiratory	0.745
Toxicity pathways		
9.	NR-AR	---
10.	NR-AR-LBD	---
11.	NR-Aromatase	---
12.	NR-ER	---
13.	NR-ER-LBD	---
14.	NR-PPAR-gamma	---
15.	SR-MMP	---
16.	SR-p53	---
Toxicophore rules		
17.	Acute toxicity rule	0
18.	Genotoxic-carcinogenicity rule	3
19.	Non genotoxic carcinogenicity rule	1
20.	Skin sensitization rule	1
21.	Non-biodegradable rule	0
22.	SURE-CHEMBL rule	0
23.	FAF-drugs4 rule	1

A score of 0 indicates no toxic structural alert; scores greater than zero indicate the detection of matched toxic fragments. For categorical toxic endpoints, the prediction probability values are converted into six risk grades: 0–0.1 (---), higher probability values correspond to greater toxic risk.

**Table 2:** Therapeutic efficacy of Cepharanthine and gemcitabine against pancreatic carcinoma AsPC-1 mouse models.

Treatment Groups	Number of Mouse	Tumor Weight (g)	Inhibition Rate (%)
		Mean ± SD	Mean ± SD
Control	5	0.8855 ± 0.2091	-
Gemcitabine	5	0.4896 ± 0.0824	44.7 ± 10.4 ^###^
Cepharanthine	5	0.5773 ± 0.0847	34.8 ± 10.7 ^##^
GEM + CEP (10 mg/kg)	5	0.3457 ± 0.0331	61.0 ± 4.2 ^###^ ***
GEM + CEP (20 mg/kg)	5	0.2103 ± 0.1549	76.3 ± 19.6 ^###^ ***

Inhibition rate (%) = (control groups − treatment groups)/control groups × 100%. ^##^ represents *p* < 0.05 when compared each group with the control while ^###^, *p* < 0.01. *** indicates *p* < 0.01 compared with the gemcitabine-treated group.

## Data Availability

The data that support the findings of this study are available from the corresponding author upon reasonable request.

## Supplementary Materials

The following supporting information can be downloaded at: https://www.mdpi.com/article/10.3390/life16091560/s1.
