# Supplementary material for: Targeted Cepharanthine-Mediated Dual-Pathway Inhibition Sensitizes Pancreatic Cancer to Gemcitabine
Source: Life (Basel). 2026 Sep 17;16(9):1560. doi: 10.3390/life16091560 (PMC13608653; doi:10.3390/life16091560)
Supplement: Supplementary file 1 [file life-16-01560-s001.zip › Table S1. Parameters for molecular docking.pdf]

Table S1. Parameters for molecular docking

Gene Name	PDB ID	Selected chain	Grid-box center (X, Y, Z)	Box dimension (grid points)	Grid spacing (Å)
AKT1	1h10	A	(21.76, 14.44, 9.63)	39.98 × 38.55 × 42.83	0.375
MTOR	1aue	A, B	(20.59, 3.85, 29.84)	52.48 × 41.32 × 65.88	0.375
PIK3CA	2enq	A	(-2.22, -2.88, 0.10)	44.13 × 64.36 × 36.78	0.375
PIK3R1	1pks	A	(6.87, -6.22, -4.83)	36.11 × 36.11 × 36.11	0.375
PD-L1	5n2d	A	(-3.787, 54.285, -32.816)	29.16 × 56.31 × 31.17	0.375
PD-1	3rrq	A	(-18.552, 16.592, 2.964)	22.50× 31.50× 47.25	0.375

Note: Box dimension represents the number of grid points. The actual spatial size (Å) was calculated as grid points multiplied by the grid spacing (0.375 Å). Selected chain indicates the protein chain(s) retained for molecular docking.
