# Supplementary material for: Targeted Cepharanthine-Mediated Dual-Pathway Inhibition Sensitizes Pancreatic Cancer to Gemcitabine
Source: Life (Basel). 2026 Sep 17;16(9):1560. doi: 10.3390/life16091560 (PMC13608653; doi:10.3390/life16091560)

Protein marker: WJ107 (Yasen)

A. represents all Western blot results in Figure 1.

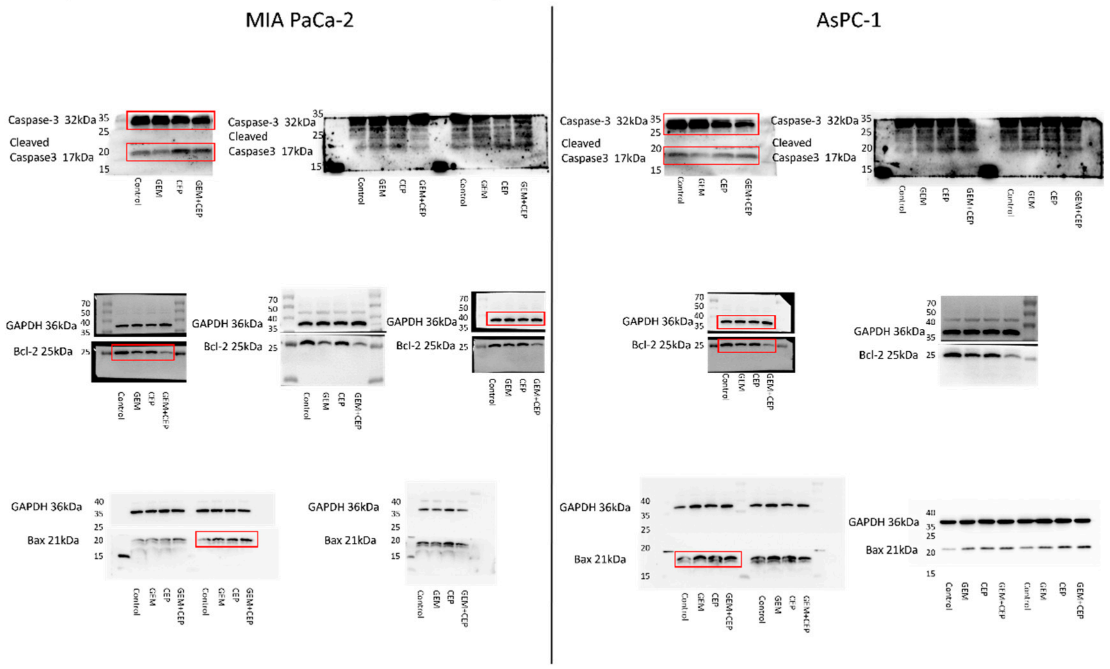

Protein marker: KGB3108-250 (KeyGEN BioTECH)

B. represents all Western blot results in Figure 4.

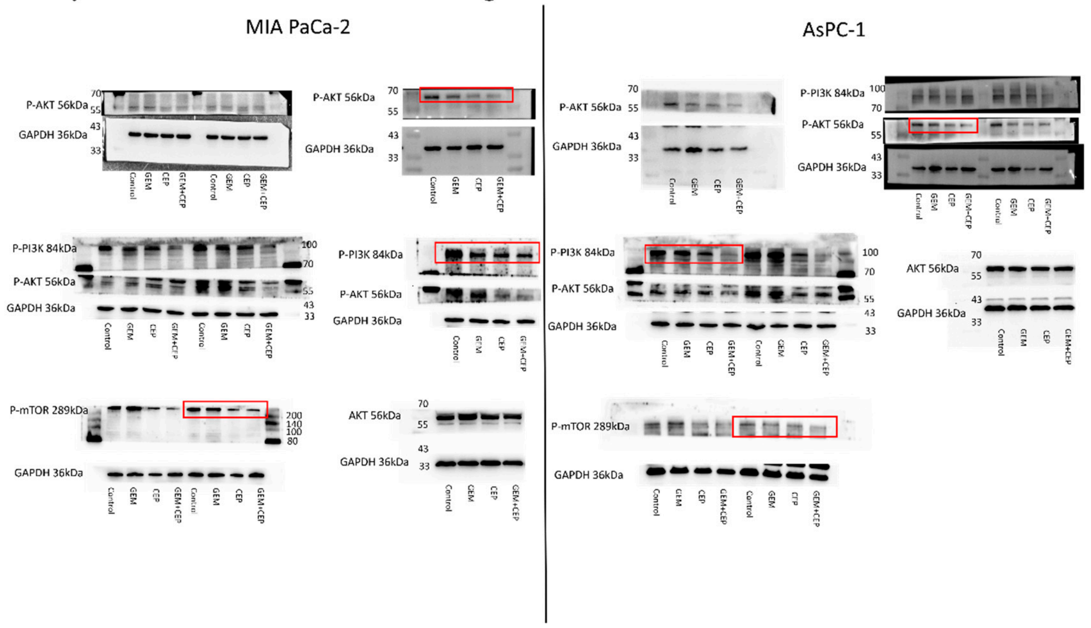

C. represents all Western blot results in Figure 4.

Protein marker: WJ107 (Yasen)

MIA PaCa-2

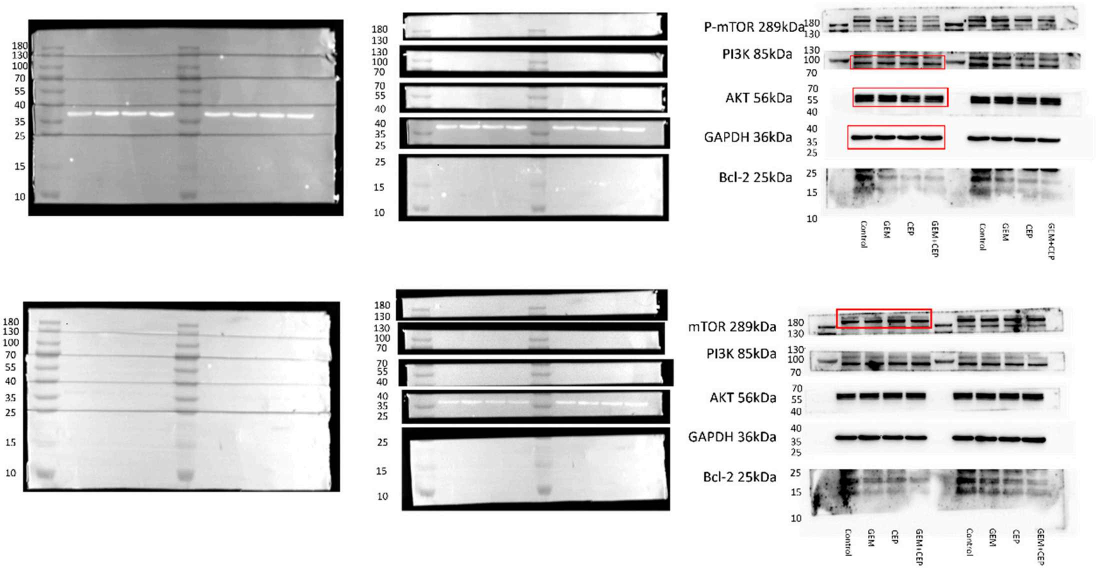

D. represents all Western blot results in Figure 4.

Protein marker: WJ107 (Yasen)

AsPC-1

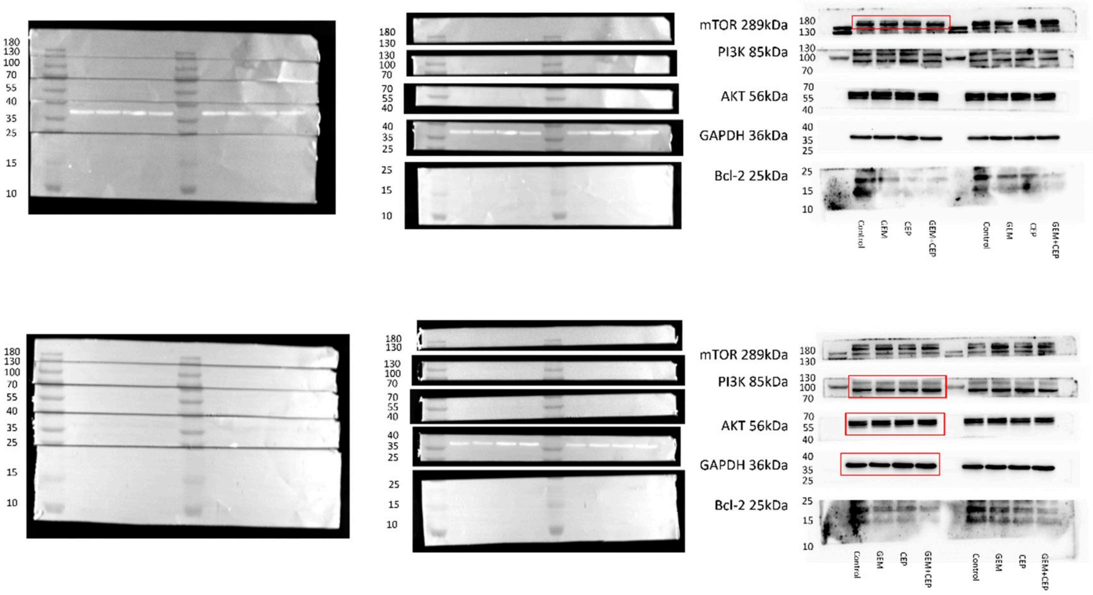

E. represents all Western blot results in Figure 5.

Protein marker: ZS-PR24002 (ZS GenTech)

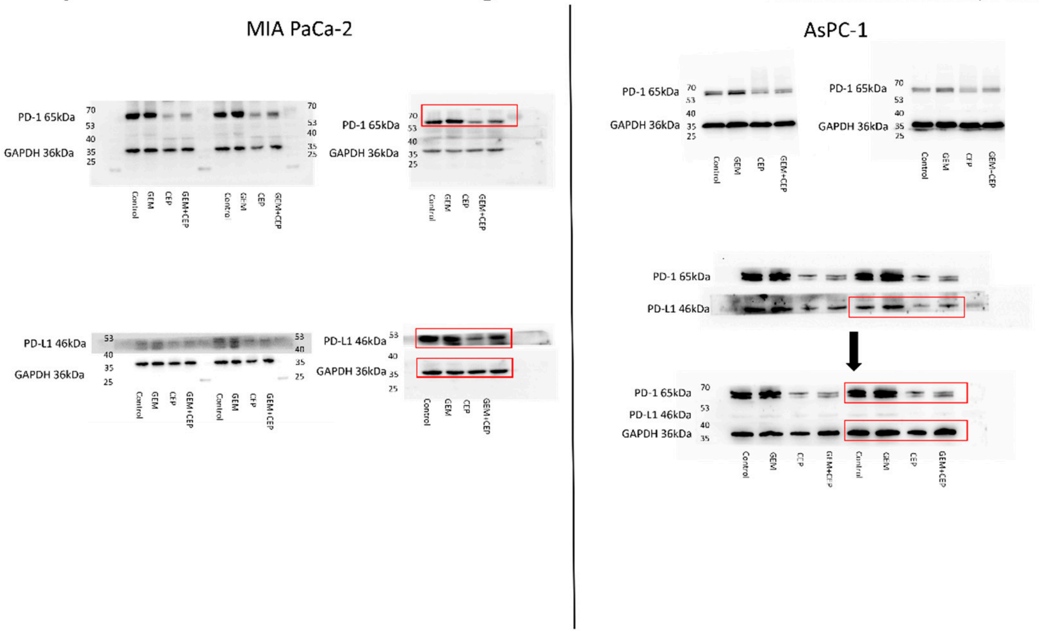

F. represents all Western blot results in Figure 6.

Protein marker: WJ107 (Yasen)

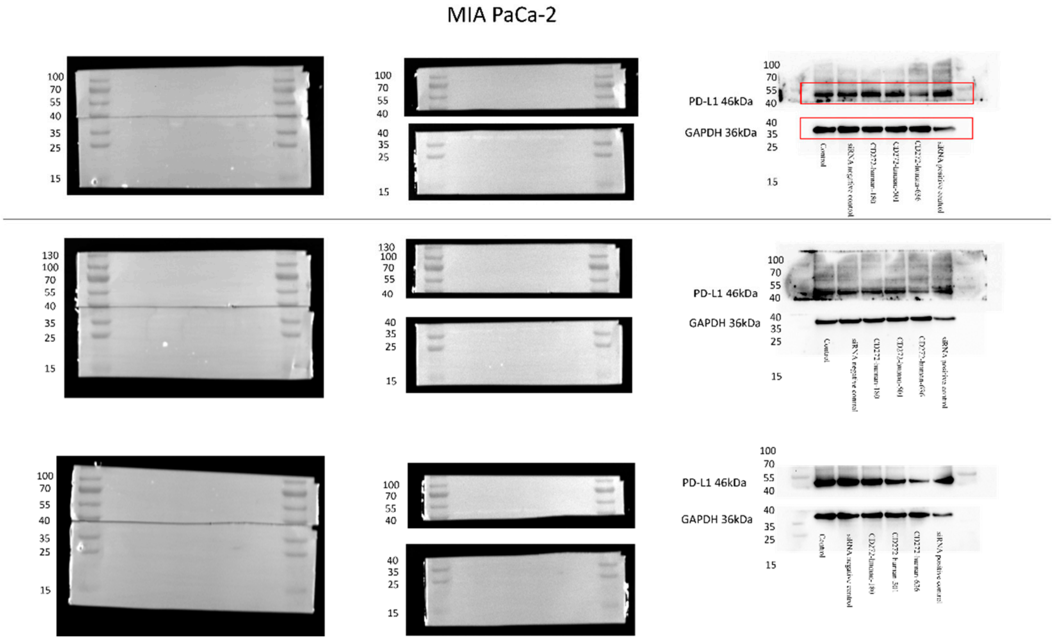

G. represents all Western blot results in Figure 6.

Protein marker: WJ107 (Yasen)

AsPC-1

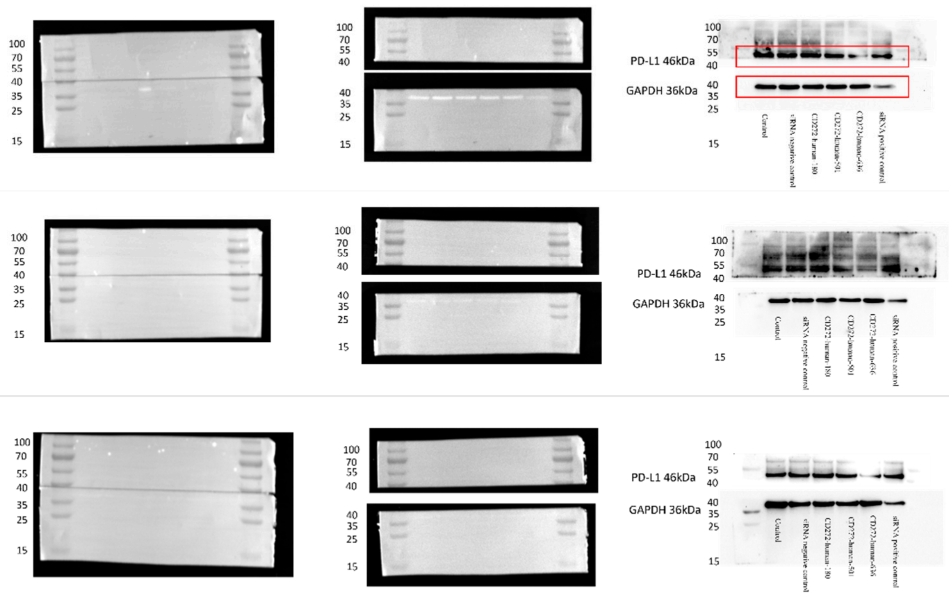

Supplement: Supplementary file 1 [file life-16-01560-s001.zip › Western Blot File.pdf]
